# Structural unification of diverse transmembrane acyltransferases reveals a conserved fold for the transmembrane acyl transferase (TmAT) superfamily

**DOI:** 10.1016/j.jbc.2025.110546

**Published:** 2025-08-05

**Authors:** Bethan E. Kinniment-Williams, Vytaute Jurgeleviciute, Daniel T. West, Reyme Herman, James N. Blaza, Marjan W. van der Woude, Gavin H. Thomas

**Affiliations:** 1 Hull York Medical School, University of York, York, UK; 2 York Biomedical Research Institute, University of York, York, UK; 3 York Structural Biology Laboratory, Department of Chemistry, University of York, York, UK; 4 Department of Biology, University of York, York, UK

## Abstract

The movement of acyl groups across biological membranes is essential for many cellular processes. One major family of proteins catalysing this reaction are the acyl transferase family 3 (AT3) proteins, which form a pore to allow acyl-CoA to penetrate the membrane for transfer onto an extracytosolic acceptor molecule. Recent structures of the sequence-unrelated human heparan-α-glucosaminide *N*-acetyltransferase (HGSNAT) support a similar transmembrane acyl-group transfer mechanism. Here we demonstrate that both protein families contain a conserved 10-transmembrane helical fold with high structural and detectable sequence conservation around the acyl-CoA pore, supporting the previously proposed Transmembrane Acyl Transferase (TmAT) protein superfamily. In addition, we identify TmAT proteins, including the human Golgi sialate-O-acetyltransferase (CASD1), the human/fungal PIG-W/GWT1 enzymes and the bacterial vancomycin resistance protein VanTG, where the TmAT domain’s function has been largely unrecognised. We conclude that the TmAT fold represents an ancient architecture for transmembrane acyl-group transfer with important roles in the dynamic modification of glycans in diverse processes across the three domains of life.

The modification of sugars *via* O-acylation is used across biology to alter the structure and properties of glycans (1, 2, 3) and impacts processes ranging from glycan degradation in the human lysosome (4, 5, 6) to evasion of immune and viral attack on fungal and bacterial pathogens (7, 8, 9, 10). While many acylations occur in the cytosol using soluble enzymes that have access to acyl-CoA pools as acyl-donors (11), there are instances where the sugar is not modified until it has been moved outside of the cytosol into another cellular compartment or surface of the cell (12, 13). Members of the Acyltransferase-3 (AT3)/Putative Acetyl-CoA Transporter (ATAT) family (TCDB 9.B.97) (InterPro IPR002656) of membrane-bound proteins facilitate the extra-cytosolic modification of some of these sugars (2). For example, O-acetylation of the oligopolysaccharide (OPS) in *Shigella flexneri* is catalysed by AT3 proteins, including OacB and Oac, resulting in a serotype conversion which is a recognised virulence determinant (14, 15, 16, 17). In *Staphylococcus aureus* and *S. epidermidis* IcaC is important for the development of poly-*N*-acetyl-glucosamine (PNAG)-derived biofilms and has been suggested to O-succinylate the polysaccharide (18). While both OacB and IcaC are thought to be standalone AT3 proteins, some members of the AT3 family are fused to a second soluble domain. A commonly identified fusion involves a SGNH hydrolase domain which is believed to participate in the transfer of the acyl-group from the AT3 domain to the extra-cytoplasmic carbohydrate acceptor (1, 2, 13, 19). An example of an AT3-SGNH fusion is the widely found peptidoglycan O-acetylase, OatA, again found in *S. aureus*. The O-acetylation of the GlcNAc sugar within peptidoglycan occurs after it has been exported out of the cytosol and confers the important phenotype to the cell of resistance to host lysozyme (12, 13). We characterized another example of an AT3-SGNH fusion in the form of the *Salmonella* LPS O-acetylase OafB (1) which confers resistance to bacteriophage infection. Using computational methods, we identified a stable 10 transmembrane helix (TMH) core structure which contains a pore that accommodates the acetyl-CoA (AcCoA) donor (1). Fused AT3 proteins are also found across taxa including eukaryotic proteins such as the *Drosophila melanogaster* Drop Dead protein (DRD) (20) and the *Caenorhabditis elegans* nose fluoxetine resistance proteins (21). Unlike their bacterial counterparts, these proteins have an NRF domain fused to the N-terminus of the AT3 domain.

The recent structures of the human membrane-bound heparan-α-glucosaminide *N*-acetyltransferase (HGSNAT) protein (4, 5, 6) provide exciting and important information about the intramembrane transfer of acyl groups onto sugars in a protein that, based on sequence analysis, has been considered unrelated to the AT3 family of acyltransferases. HGSNAT acetylates heparan sulfate for its subsequent breakdown in the lysosome, and loss of function leads to the accumulation of heparan sulfate in the lysosome, causing diseases such as Sanfilippo syndrome (22). In what appears to be a strikingly similar process to OafB, the HGSNAT protein binds acetyl-CoA on the cytosolic face, with the protein creating a pore for it to insert into the membrane and present the acyl-group to a catalytic site on the opposite side for movement onto an extracytosolic sugar. The HGSNAT protein also contains a second domain (called ⍺-HGSNAT), although this luminal domain is structurally distinct from OafB’s SGNH domain and is cleaved after synthesis but stays associated with the 10 TMH membrane domain (called β-HGSNAT) through a single TMH. In two of these recent publications (4, 5), the AT3 proteins are not mentioned as a related family of membrane-bound acyltransferases. Additionally, Xu *et al.* (4) report finding no structural matches to HGSNAT in their DALI search of the PDB due to a lack of solved structures; However, their search did not extend to the AlphaFold database, which includes predicted structures.

Despite Xu *et al.* (4) and Zhao *et al.* (5) finding no structural similarities to HGSNAT, the Transporter Classification Database (TCDB) places OafB and HGSNAT into two different families (9.B.67 AT3 and 9.B.169 HGSNAT/YeiB, respectively), but importantly groups them into the same superfamily which is known as the Transmembrane Acyl Transferase (TmAT) superfamily (23). The third paper by Navratna *et al.* (6) recognizes the TmAT superfamily, noting their structure of HGSNAT as the first within this superfamily. They highlight the similar architecture between the transmembrane domains of an AT3, OafB, and HGSNAT but ultimately conclude that the structures are not homologous. In this analysis, we present the first evidence that there is clear structural homology across the TmAT superfamily, including homology between OafB and HGSNAT. We also undertake a detailed analysis of a larger grouping of membrane-bound proteins from several distinct families, enabling us to refine the membership of the Acyl_transf_3 Clan in Interpro to include important unrecognised members, enabling us to define conserved sequence characteristics of the TmAT fold, which cluster around their unique membrane-embedded acyl-CoA binding site. As this analysis confirms at a structural level the proposed TmAT superfamily (23), we suggest this name is adopted more widely to represent proteins with this fold found in biology.

## Results and discussion

### Proteins in the TmAT superfamily contain a core conserved 10 TMH fold

**Figure 1:**
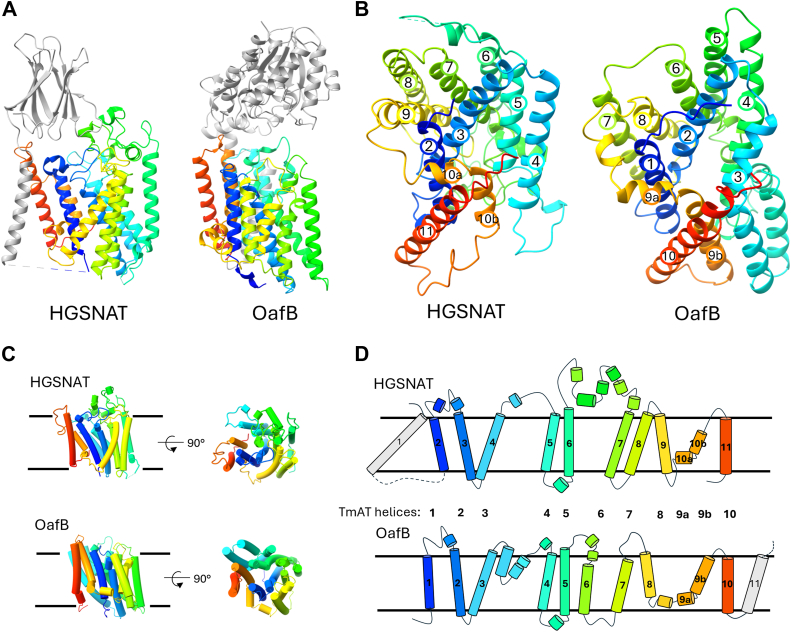
**Conservation of a core 10-TMH TmAT protein fold**. *A*, side view of HGSNAT and OafB showing their additional fused domains and helices (*grey*). *B*, cytosolic view of the core membrane fold from HGSNAT and OafB. *C* and *D*, cartoon representation of the helices of HGSNAT and OafB illustrating common elements and structural organization, where *dotted lines* represent additional structures. Proteins are all colored with rainbow coloring (N-terminus to C-terminus). HGSNAT uses PDB code 8JKV and OafB the AlphaFold model for OafB (UniProt ID A0A0H2WM30), both are apo-states without acetyl-CoA bound.

To assess whether the membrane domain architecture is homologous within the proposed TmAT superfamily, we first compared the structural similarity of the best structurally characterized AT3 protein, OafB, to the human HGSNAT using DALI tools (24, 25). As both proteins contain additional non-homologous domains (Fig. 1*A*), they were excluded from the analysis. A common structure is observed for both membrane domains (Fig. 1, *B*–*D*), which includes core helices 1 to 4 of OafB (2–5 of HGSNAT) which are involved in acyl-CoA binding, the scaffolding helices 5 to 8 of OafB (6–9 of HGSNAT), which sit ‘behind’ the core helices, and the final two helices, 9 to 10 of OafB (10–11 of HGSNAT) the first of which is an unusually bent helix that closes one side of the acyl-CoA binding site (4, 5, 6). The Z score and RMSD between the 10 TM membrane domains of OafB and HGSNAT are 13.2 and 5.0 Å with 73% coverage, supporting the hypothesis that these proteins are structurally related (24, 25). To test this idea more rigorously, we widened the analysis to 16 additional members from each of the two TCDB-defined families that contain OafB and HGSNAT (9.B.67 AT3 and 9.B.169 HGSNAT/YeiB) with representatives from bacteria, archaea, and eukaryotes. A DALI “all-against-all” comparison was used to demonstrate conservation of the fold across all examined members (Figs. S1–S4, Tables S1, and S2). In addition, using Pfam HMMs for either AT3 (PF01757) or HGSNAT (PF07786) as search queries, members of the reciprocal families were detected, strongly supporting our initial structure-based observation of homology (Table S3).

### Expanding the TmAT superfamily

Having established the structural similarity of the TmAT superfamily as defined by the TCBD, we also then critically assessed the structural and sequence similarities within the Pfam Acyl_transf_3 Clan (CL0316) that contains 9 Pfam families, including AT3 (PF01757) and HGSNAT-cat (PF07786), that are defined as having a shared evolutionary origin (Table S4) (26).

Using our DALI-based searches, we also discovered an additional Pfam family with a similar fold, the GWT1/PIGW family of membrane-bound acyltransferases, that we included in this analysis, and we note that during revision of this manuscript, the Pfam Clan was updated to include GWT1/PIGW, supporting our assertion of structural similarity.

**Figure 2:**
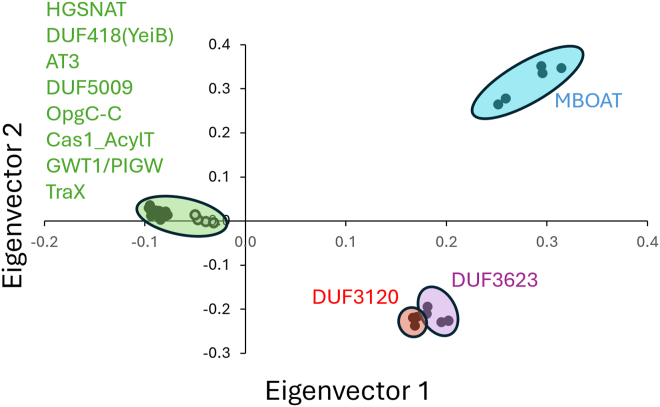
**Structural analysis of the TmAT superfamily refines the membership of the Pfam Acyl_transf_3 Clan (CL0316).** Correspondence analysis of predicted structures of representative proteins from the nine Pfam families that constitute the Pfam Acyl_transf_3 Clan (CL0316) (Table S4), plus the GWT1/PIGW (PF06423) and MBOAT family (PF03062). MBOAT was included as an out-group of a structurally unrelated membrane-bound acyltransferase. This multidimensional scaling method uses eigenvectors to separate structurally different proteins on two different axes, so the most structurally similar proteins are positioned together. Five members of each were selected for structural correspondence analysis in DALI, with full-length protein representatives from bacteria, eukaryotic, and archaea where possible (see Figs. S5, S6 and Table S6). Colors represent the different structural groups we define from the analysis. TraX proteins are depicted by an unfilled circle to highlight that they are borderline members of the TmAT superfamily.

A DALI-based correspondence analysis of the structural similarity of these proteins (Table S5) strongly supports seven of the nine original families as being truly homologous (Fig. 2). At the sequence level, searches using family-specific HMMs found hits between AT3 and HGSNAT and also DUF5009, DUF418, OpgC-C, and TraX families within the clan (Table S3). Two of the original families included in the clan, PF11318 (DUF3120) and PF12291 (DUF3623), are significantly shorter and have no shared structural organization with the TmAT fold, and we consider their membership in the TmAT superfamily is not supported; and they have now subsequently been removed from the updated Clan. The TraX family (PF05857) is the most divergent of the protein families that we considered to have the TmAT fold, lacking the characteristic broken ninth TMH and the 10th TMH. This divergence might be explained as this protein is known biologically to be required for the acetylation of a protein substrate (27), the bacterial F-pilin protein, so it differs from the carbohydrate acceptors that are usually seen for TmAT proteins. In this analysis, we also included control proteins from the MBOAT family of membrane-bound acyltransferases, such as bacterial DltB and human Hedgehog acyltransferase (HHAT) (28, 29), which are clearly structurally distinct from the TmAT fold (Fig. 2), as previously described (1).

During work for this analysis, the structure of another membrane-bound acyltransferase of the GWT1/PIGW protein family was solved (30), which our structure-based assignment includes as being within the TmAT superfamily (Fig. 2). We could not support this with our sequence-based HMMs (Table S3); however, the updated Pfam Clan (CL0316) does find a significant match between HGSNAT and GWT1/PIGW, supporting membership in the TmAT superfamily.

### The most highly conserved sequence and structural elements of the TmAT superfamily form the acyl-CoA binding site

**Figure 3:**
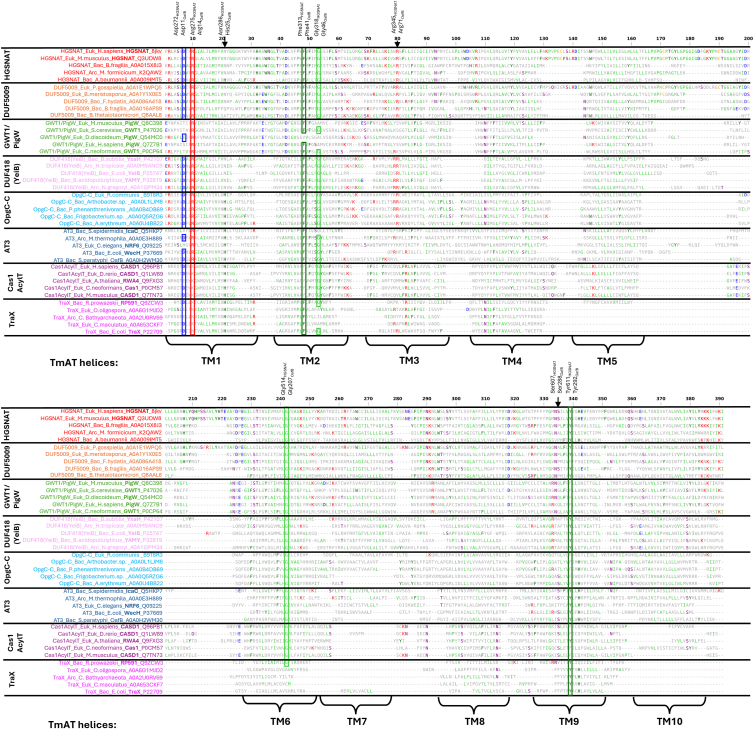
**Structural alignment of the TmAT superfamily defines the core conserved residues**. Structure-based alignment (compared to HGSNAT 8JKV) produced in DALI, showing diverse representatives of all Pfam Acyl_transf_3 Clan (CL0316) members containing the TmAT domain (see Fig. 2) and the additional PigW/GWT1 family found by a DALI search. Regions with no structural/sequence homology are not expanded, so only structurally equivalent positions to HGSNAT (8JKV) are shown. It is important to note that some of the proteins appear not to have a TM5 or TM7, but this is due to poor structural alignment with other TmAT members and not because they lack a TM5 or 7. The most common amino acid at any given position is coloured according to aromaticity (*dark green*) (Phe, His, Trp, Tyr), positive charge (*red*) (Arg, Lys), negative charge (*blue*) (Glu, Asp), polar uncharged side chains (*purple*) (Ser, Thr, Gln, Asn), hydrophobic side chains that are not aromatic (Gly, Leu, Ile, Val, Pro, Met, Ala), and sulfur-containing amino acid (*orange*) (Cys). Boxed residues are those that are conserved, or contain conservative substitutions (Phe↔Tyr, Arg↔Lys, Asp↔Glu), across the alignment. Additional partially conserved residues discussed in the text are marked with an *arrow*. See Fig. S14 for secondary structure alignment.

While we have confirmed the structural homology of the TmAT superfamily across its two constituent TCDB families and refined the Pfam Clan with the same fold, we next sought to use our structural alignments to uncover if there was any sequence conservation across these functionally diverse acyltransferase proteins (Fig. 3). We aligned the core 10 TMH for our 50 example proteins (Table S5) from the ten candidate families of protein based on the Pfam Clan plus GWT1 (Fig. S7), which separate on an unrooted tree into clear families (Fig. S8). We note that the DUF5009 proteins intermix with the HGSNAT clade, as seen in our structure-based clustering (Fig. S6), suggesting strongly that these proteins are in fact orthologous proteins, and while there is no experimental confirmation of the function of any DUF5009 protein, they are likely to be closely related to that of HGSNAT. Also, we note that the DUF3623 and DUF3120, which sit within the existing Pfam Clan, are no more distantly related to the other members of the superfamily, despite having an entirely different predicted protein fold, demonstrating the weakness of sequence-only based approaches for largely integral membrane proteins that have a high hydrophobic amino acid composition (31, 32).

We then compare these findings to the limited number of studies where mutagenesis has been used to study the function of proteins in the TmAT superfamily. Within our alignments of the members of the superfamily (Fig. 3), there were six positions that were highly conserved (>3 average BLOSUM62 score (Fig. S9)), with four occurring in TMH 1 to 3 and the others in TMH six and TMH 9, consistent with the most conserved structural features of the fold (Fig. S10). We refer to these residues as TMH#-Xxx, where TMH# is the TMH harbouring the residue and Xxx is the amino acid. The corresponding residues in HGSNAT and OafB are found in Table S7.

Working through the proteins, the TMH one contains a conserved DxxR motif with both residues positioned at the cytosolic side of the protein. While the Asp (TMH1-Asp) in this motif is essential for the function of HGSNAT, it appears to be dispensable for the function of the AT3 protein, OacB (Asp44) (4, 14) (mutated residues are shown in Fig. S11). However, the Arg residue (TMH1-Arg) is essential for the function of HGSNAT, OafB and OacB (Arg47) (4, 7, 14). It is of note that in two of the Pfam families included in the Acyl_transf_3 Clan, Cas1_AcylT and TraX, the Arg is usually replaced by a Lys. Moving into TMH2, there is a conserved FxxxxG motif, where the Phe (TMH2-Phe) has been shown to be important for the activity of HGSNAT and the AT3 protein, OatA (Phe52) (4, 13). However, the constituent conserved Gly (TMH2-Gly) was found to be non-essential in OatA (13), and no mutagenesis studies were conducted on this residue in HGSNAT. In AT3 proteins, the widely recognized TMH3 RxxR motif has been shown to be important for the function of OafB, OafA, Oac, and OacB (2, 4, 8, 13, 14, 15, 16). The first Arg (TMH3-Arg) of this motif is conserved in most members of the TmAT superfamily and its mutation in HGSNAT causes a significant loss of activity (2, 4, 8, 13, 14, 15, 16). An additional highly conserved Gly (TMH6-Gly) was identified in TMH6 and in HGSNAT, a missense mutation of this residue (G514 E) is found in patients with Sanfilippo type C disease (7, 33). The final conserved residue is a Tyr in the bent TMH 9 (TMH9-Tyr). Although this position has not been investigated *via* mutation in HGSNAT, Tyr611 surrounds the beta-mercapto-ethylamine of acetyl-CoA (4), and the equivalent residue in OatA has been shown to be important for protein function (13). In AT3 proteins, this Tyr is part of the SxxxY motif in TMH 9 (1) which is also conserved in the human HGSNAT protein. However, the Ser (TMH9-Ser) is not conserved across all members of the superfamily, and the equivalent residue has been shown to be non-critical for the function of OatA (Ser310) (13).

To support the structure-based identification of key residues, we also examined sequence-based alignments using Pfam HMMs for each family (Fig. S12). The identification of the key residues within each Pfam HMM was determined by the stacked sequence logos from DALI (Fig. S13). We find similar conservation of the key residues mentioned above (Fig. 3), although note that the Pfam HMM for the DUF418 (YeiB) is very short and only includes the end of the protein and that GWT1 appears to be the most divergent from the TmAT profile in lacking a DxxR motif. Using these HMMs in hmmsearch, we were able to find consistent matches between AT3, HGSNAT, DUF5009, DUF418 (YeiB), and OpgC (Table S3). TraX is found using the AT3 HMM but not others, supporting its more distant structural similarity to the other members of the superfamily.

Despite not finding reciprocal hits for Cas1_AcylT and GWT1/PigW searching with other TmAT family members (Table S3), we note that the updated Pfam clan (CL0316) reports a match with an E-value of 2.1 × 10^−^4 (just beyond our cutoff) showing that even GWT1 has some sequence similarity and not purely structural similarity to HGSNAT. Also, an independent study proposed that CAS1 and AT3 are sequence related through conservation of the Arg/Lys and His in TMH1 and the Arg in TM3, broadening the sequence-based support for the superfamily (34).

**Figure 4:**
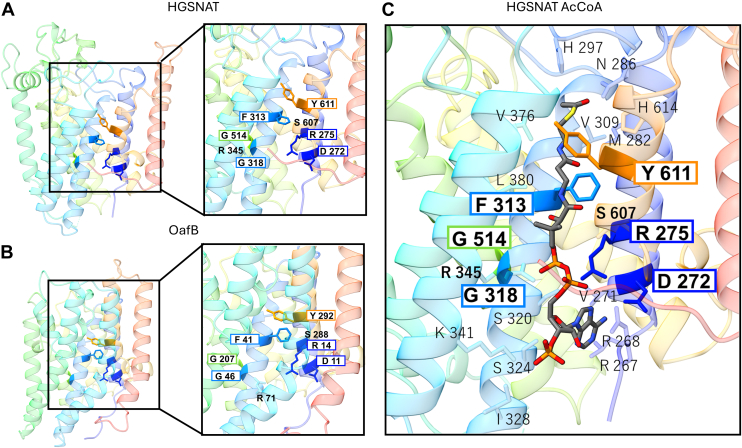
**The conserved residues of the TmAT superfamily form the acyl-CoA binding site**. Conserved residues in the TmAT superfamily superimposed on (*A*) the 10 TM domain of HGSNAT (8JKV) and (*B*) the 10 TM domain of OafB, illustrating their common arrangement and in (*C*) with acetyl-CoA bound in HGSNAT (8JL1) illustrating their role in acyl-CoA recognition. The seven highly conserved residues are boxed and in bold text, with the additional partially conserved residues, Ser607 (TMH9-Ser) and Arg345 (TMH3-Arg) (see text) in bold, but unboxed. TMH1 is shaded in *dark blue*, TMH2 in *blue*, TMH3 in *cyan*, TMH6 in *green*, and TMH9 in *orange*. The residues shown in a smaller text represent the residues interacting with acetyl-CoA according to the PDB structure 8JKV (4). Note that the F313 and R345 structurally reorient during acetyl-CoA binding (4).

Given the newly identified sequence and structural features of the TmAT superfamily, we assessed how these most conserved elements might be involved in enzyme function. Strikingly, but perhaps not surprisingly, the most conserved elements of the TmAT proteins are involved in the binding of the conserved acyl-CoA donor molecule (Fig. 4). Building on prior work on AT3 proteins, which predicted the OafB acyl-CoA binding site (1), the new structures of HGSNAT (4, 5, 6) provide the precise location of the acetyl-CoA binding site. This location is largely consistent with the location predicted by molecular dynamics simulations for OafB (1).

The conserved amino acids form what appears to be an equivalent binding site formed by the same helices in HGSNAT (TM2-5, TM10, and TM11) and in AT3 (TM1-4, TM9, and TM10) (Fig. 4). Acetyl-CoA binds in an extended conformation to enable it to enter the protein from the cytosol and penetrate through the membrane to the enzyme’s catalytic site on the extra-cytosolic face (Fig. 4). The conserved residues in the TmAT superfamily play key roles in the interactions of the protein with the full length of the acyl-CoA molecule in its binding site, with the majority interacting with the “common” adenosine and the 4-phosphate pantothenic acid moiety of CoA found in all acyl-CoA substrates (Fig. 4). These are on the cytosolic side and should be the most conserved elements given that our alignment includes TmAT superfamily members that use succinyl-CoA and other longer chain acyl-CoA as acyl-donors (18, 30, 35), where the structure of the acyl-donor varies beyond the thioester bond. Interestingly, the “family-specific” conserved residues are found to line the acyl-CoA binding site further towards the extracytosolic side (Fig. 4). This includes an important Asn residue in HGSNAT further along TMH 1, which in AT3 proteins is found as a His residue in a family-specific DxxRx_10_H motif (1). Both of these residues are important for function and have been proposed in some cases to be involved in catalysis following acyl-CoA binding (4, 13).

Taken together, these analyses of the shared structural and sequence conservation of proteins in the AT3 and YeiB/HGSNAT families entirely support the concept of them forming the TmAT superfamily and having a conserved evolutionary ancestry.

### Assessment of new members of the TmAT superfamily

Having established the power of comparing AlphaFold-derived structural models using DALI to establish the evolutionary basis of the TmAT superfamily, we looked deeper into the results to seek out further important proteins, that are either currently not classified into the two constituent families in the TCBD or are in Pfam families not currently included in the Acyl_transf_3 Clan (CL0316). Using the bacterial AT3 protein OafB as a query against the Human AlphaFold database in DALI, HGSNAT is returned as a strong match (UniProt ID: Q68CP4, Z score: 13.1, RMSD: 4.7 with 37% coverage) in addition to two other interesting proteins, the CASD1 protein (UniProt ID: Q96PB1, Z score: 17.1, RMSD: 4.5 with 34% coverage), and PIGW/GWT1 protein (UniProt ID: Q7Z7B1, Z score: 12.1, RMSD: 4.6 with 48% coverage), both of which are discussed below.

## PIGW/GWT1

**Figure 5:**
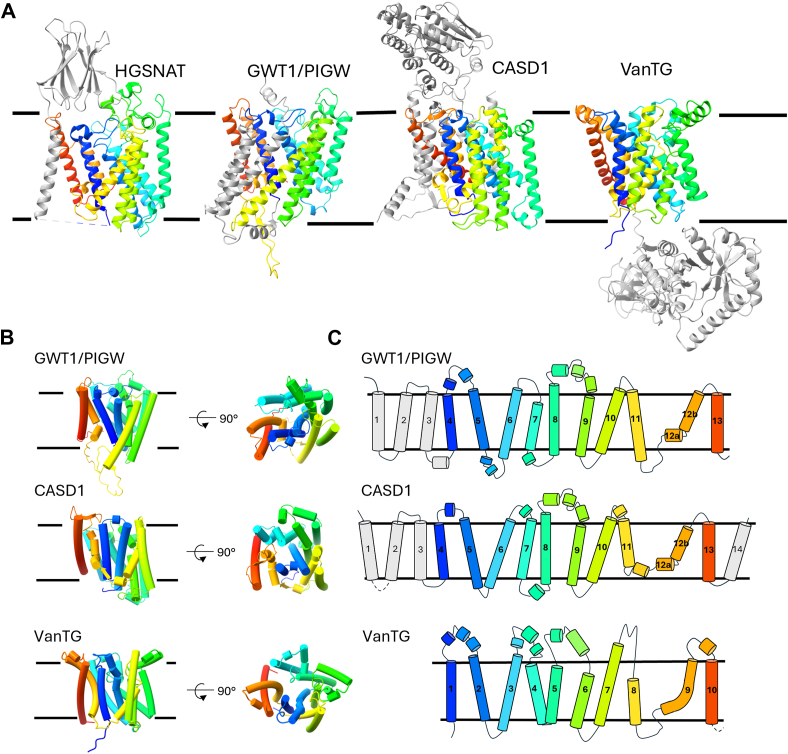
**The presence of the core TmAT fold implies previously unrecognised additional biochemical functions in diverse biologically characterised proteins.***A*, Side view of HGSNAT, GWT1/PIGW, CASD1 and VanTG showing their additional fused domains and helices (*grey*). *B*, and *C*, Cartoon representation of the helices of HGSNAT, GWT1/PIGW, CASD1, and VanTG illustrating common elements and structural organisation, where *dotted lines* represent additional structures. Proteins are all coloured with rainbow colouring (N-terminus to C-terminus). The following PDB or Alphafold IDs are used: HGSNAT (PDB ID: 8JKV), GWT1/PIGW (UniProt ID: Q7Z7B1), CASD1 (UniProt ID: Q96PB1), VanTG (UniProt ID: Q186I3); all are apo-states without acyl-CoA bound.

The PIGW/GWT1 proteins (PF06423, IPR009447), we previously discussed, are not recognized in either the Pfam Acyl_transf_3 Clan (CL0316) or the TCDB TmAT superfamily, yet our structural evidence (Figs. 2 and 3) supports their placement within the TmAT superfamily. The newly solved structure of the fungal GWT1 protein confirms this hypothesis by revealing the characteristic TmAT superfamily fold (30). (Fig. 5) and closely resembles the AlphaFold model (Figs. S15 and S16). Their structure-based comparisons, like ours (Table S6), suggest that GWT1 is more closely related to the HGSNAT family than to other members of the TmAT superfamily. Although uniquely, the GWT1 proteins lack the D of the DxxR motif in TM1, and the catalytic histidine seen in HGSNAT is absent (30). Biologically, these proteins are interesting TmAT members as they catalyze the addition of an acyl-group onto an inositol sugar acceptor in an early stage of the synthesis of glycosylphosphatidylinositol (GPI) (36, 37). Mutations in the human gene result in the loss of inositol acetylation, which reduces levels of GPI-anchored proteins and leads to West syndrome and hyperphosphatasia with mental retardation syndrome (HPMRS, also known as Mabry syndrome).

In fungi the same protein, called GWT1, is required for the synthesis of their complex cell envelope and has been targeted in the development of novel anti-fungal (38) and anti-parasitic drugs (39). Our inclusion of these proteins in the TmAT superfamily has a functional consequence for these proteins, which is supported completely by the new structure. This relates to the spatial localization of this reaction in the GPI biosynthesis pathway as the inositol acylation step occurs before a mannosylation step (36) that is catalyzed on the luminal side of the ER membrane. Hence our structural model and the experimental structure now suggest that the immediately preceding inositol acylation step would also likely occur on the luminal side of the ER membrane using cytosolic palmitoyl-CoA as the acyl-donor, rather than requiring there to be a pool of palmitoyl-CoA within the ER lumen as proposed by Sagane *et al.* (40) GWT1 is also interesting as the acyl-group donor is much longer than other TmAT proteins (palmitoyl is C16) and the structure reveals that while the CoA part of the palmitoyl-CoA binds in a similar way to other TmAT proteins (30), the longer fatty acid attached to the thiol group bends back into a broader channel in the protein, which is known to be able to accommodate acyl-CoA’s as short as C10 *in vitro* (41), suggesting more flexibility, which is borne out by some sequence differences in the GWT1/PIG-W proteins compared to other TmAT superfamily members (Fig. 3).

## CASD1

The human sialate O-acetyltransferase CASD1 (Fig. 5) is an important Golgi protein that is essential for O-acetylation of sialic acids during the maturation of glycans and a potential target for preventing cancer progression (42). While it has been previously noted to be a homologue of the *Cryptococcus neoformans* Cas1p protein that O-acetylates yeast glycans (43), the significance of it being a two-domain protein that includes a TmAT domain has not been fully appreciated. Like the bacterial OafB protein, CASD1 also contains an SGNH transferase domain, which in this case would be located in the Golgi lumen. In OafB, the periplasmic SGNH domain catalyzes the final step in the acetylation reaction, accepting the acetyl-group from the AT3 domain and adding it to the sugar residues in the LPS (1, 2). Studies on the human CASD1 protein have largely focused on this SGNH domain (43), which is N-terminal in CASD1, while the C-terminal TmAT domain has not been studied biochemically. The recognition of this second domain opens the possibility that acetyl-CoA does not have to be transported into the Golgi, as is currently thought (3), as the acetyl group could be delivered across the membrane to the luminal SGNH domain *via* the TmAT domain, much like in the bacterial OafB protein (1). Much of the evidence for a Golgi acetyl-CoA transporter comes from elegant work by Varki & Diaz (44), who took purified Golgi and followed the fate of acetyl-CoA radiolabeled on the acetate. Strikingly, they found that most of this label (75–85%) was found on O-acetylated sialic acids in the Golgi, which supports the idea that there is a specific connection between the acetyl-CoA pool and the sialic acid acceptors in the two cellular compartments that could be mediated by the two domains of CASD1. This would ameliorate the need for the transport of acetyl-CoA into the Golgi, which might lead to its turnover by many enzymes and not specific labelling of sialic acids. Perhaps consistent with this, the acetyltransferase activity was lost when the Golgi membrane was disrupted, suggesting delivery of the acetyl-CoA *via* the TmAT domain of CASD1 is required for efficient acetylation (45).

## VanTG

Finally, we wish to highlight VanT, an important protein implicated in the resistance of pathogenic bacteria to the clinically important antibiotic vancomycin. VanT (for example, UniProt ID Q186I3) contains two domains, an AT3 domain (classified within Pfam as AT3) fused to a cytoplasmic serine racemase domain (Fig. 5). As a known mechanism of resistance to vancomycin involves altering its binding site in the cell wall from containing a D-Ala-D-Ala structure to a D-Ala-D-Ser structure, the function of VanT has only been considered its serine racemase domain, as part of the pathway to replace D-Ala with D-serine, made from L-Ser by VanT (46, 47). However, mutations that arise both in the lab and in clinical samples that lead to high-level vancomycin resistance fall into the AT3 portion of the *vanT* gene and not the racemase portion (48). Hence, the AT3 domain itself clearly has an important function that has not been considered, but the new insights into this domain should facilitate future analyses.

### Concluding remarks

The improved knowledge of acyl-CoA binding in the TmAT superfamily, combined with insights into catalysis from the HGSNAT structures, can also now be used to shed light on other AT3 protein family members, which have important biological phenotypes but a poor understanding of biochemical function. These include, for example, the NDG-4 protein from *C. elegans* that regulates lifespan (21, 49) and the HIF-1 Hypoxia-inducible factor inhibitor Rhy-1 from *C. elegans* (50), which together are only two of the 63 AT3-containing proteins encoded in the *C. elegans* genome (50). We also note that several fungal AT3-family proteins are encoded within biosynthetic clusters of various natural products, including the cholesterol-lowering drug squalestatin S1 (51). Given that we already know that in *Streptomyces* sp. there are antibiotic biosynthesis clusters that contain AT3 proteins (2) where they are known to catalyze the final acylation step in the biosynthesis step following export across the inner membrane (52), we propose that these fungal proteins, such as *Phomopsis amygdali* Orf9 in the fusicoccin cluster (53) and *Cochliobolus lunatus* Clz18 in the Zaragozic acid A cluster (54), function in natural product biosynthesis pathways.

In conclusion, in this analysis, we demonstrated the power of structural comparisons to accurately assign members to the TmAT superfamily and distinguish subfamilies otherwise not observed at the sequence level (Fig. S8). In some cases, we can also see low levels of sequence conservation within the superfamily, but the higher resolution of structural conservation is key to this analysis, and has been used powerfully to demonstrate common origins for other membrane protein classes (55, 56). We identified important residues and a conserved 10TMH architecture that defines this TmAT superfamily, which allowed us to identify previously unrecognized proteins doing transmembrane acylation reactions of glycans and to exclude non-structurally related families. This enables us to use the exciting and important data on acyltransferase mechanism from the recent HGSNAT work (4, 5, 6) and GWT1 protein (30) to infer similar mechanisms across a broad range of protein functions from archaea to man and significantly advances our understanding of many known AT3 and other TmAT superfamily proteins.

## Experimental procedures

### Bioinformatics methods for DALI all-against-all analysis

The DALI (24, 25, 57) all against all tool was used to compare the 32 proteins included in the analysis of the TCDB (23) defined TmAT superfamily. This tool was also used to compare the 55 proteins from the nine families in the Pfam Acyl_transf_3 Clan (CL0316), the GWT1/PIG-W family and the MBOAT superfamily which helped to refine the TmAT superfamily classification. This approach was used to generate heat maps, multiple sequence alignments, structural alignments, structural dendrograms, correspondence analyses, and provided RMSD values and percentage sequence identity.

### Selection method for proteins in the two families in the TmAT superfamily represented in the TCDB

For DALI all against all analysis, we selected 16 proteins using UniProt (accessed between 08/24–07/25), or PDB if available, from the Acyl_transf_3 (PF01757), and another 16 from DUF418 (PF04235)/HGSNAT-cat (PF07768). Six eukaryotic members, five bacterial members, and five archaeal members were included for each member of the TCDB-defined TmAT superfamily (32 proteins were included in this analysis in total). Exact UniProt ID’s/PDB ID’s, along with the domain and species, can be seen for these 32 proteins in Fig. S3 (Structural dendrogram). The names follow the format as follows: ProteinFamily_Domain_Species_PROTEINNAME_UniProtID/PDBID. Protein names are in bold in Fig. S3 unless they have yet to be named/are unreviewed. To focus the alignment on the core membrane helices, additional helices at either end and additional N- or C-terminal globular domains were removed (for example, the *β*-HGSNAT domain was excluded from HGSNAT, and the C-terminal SGNH domain plus the linker was excluded from OafB). To see the exact truncations that were used in the analysis, see Table S1. Proteins were aligned in CCP4MG (58) *via* Secondary Structure Matching (SSM) to human HGSNAT (PDB ID: 8JKV). Identifying the overlap between the TmAT 10 TM in HGSNAT and other TmAT superfamily members allowed us to identify the 10 TM TmAT fold in the other TmAT superfamily members. These superpositions were then exported as PDB files and put into ChimeraX1.8, where they were cut down to the appropriate 10 TM helices.

### Selection method for proteins from the the Pfam Acyl_transf_3 clan (CL0316), GWT1/PIG-W family and MBOAT superfamily

For the DALI all against all analysis, the following selection method was used for the proteins that were included. Five proteins using UniProt (accessed between 08/24–07/25), or PDB if available, were selected from following families; Acyl_transf_3 (PF01757), DUF418 (PF04235), HGSNAT-cat (PF07768), TraX (PF05857), Cas1_AcylT (PF07779), OpgC-C (PF10129), DUF3120 (PF11318), DUF3623 (PF12291), DUF5009 (PF16401), GWT1/PIGW (PF06423), and the MBOAT (PF03062) which served as our negative control (55 proteins were included in this analysis in total). To focus the alignment on the core membrane helices, additional helices at either end and addition N- or C-terminal globular domains were removed (for example, the *β*-HGSNAT domain was excluded from HGSNAT, and C-terminal SGNH domain plus the linker was excluded from OafB). To see the exact truncations that were used in the analysis, see Table S5. For protein families with fewer than 10 TMs (*e.g.*, TraX with 9 TMs, DUF3623 and DUF3120 with 7 TMs), sequences were trimmed accordingly to nine or 7 TMs. MBOAT proteins were not trimmed to 10 TMs due to poor alignment with HGSNAT and the absence of additional domains seen in other proteins; they were instead included as an outgroup. Exact Uniprot ID’s/PDB ID’s, along with the domain and species, can be seen for these 55 proteins in Fig. S6 (Structural dendrogram). The names follow the format as follows: ProteinFamily_Domain_Species_**PROTEINNAME_UniProt ID’s/PDB ID’s. Protein names are in bold in** Fig. S6 unless they have yet to be named/are unreviewed. For this analysis we selected a mixture of bacterial, eukaryotic and archaeal members where possible. However, not all families included examples from all domains, so other domain protein members were used to make it up to 5. In general, we selected proteins reviewed in UniProt to ensure higher-quality data and reliable AlphaFold structures.

### Structure-based multiple sequence analysis (MSA)

For structure based multiple sequence alignments, all sequences were compared to the sequence of HGSNAT (PDB: 8JKV). Sequences are arranged by the family they belong to. Note that DUF3623, DUF3120 and MBOAT were excluded from the MSA analysis as they are not part of our defined TmAT superfamily. In the alignment for the TCDB-defined sequences and revised Pfam clan (Figs. 2 & 3), if an amino acid was present in > 80% of sequences at a particular position, it was boxed. Amino acids were grouped together as (Phe, Tyr, His, Trp), (Ser, Thr, Gln, Asn), (Arg, Lys), (Glu, Asp), (Cys), (Gly), (Met), (Val), (Iso), (Leu), (Ala), (Pro) Additionally, for the alignment of the revised Pfam Clan (Fig. 3), residue conservation was calculated using the BLOSUM62 substitution matrix. For each alignment column, all possible amino acid pairs were scored according to the BLOSUM62 matrix, and the total was then divided by the number of possible pairs (Fig. S9). This approach provides an estimate of conservation because more conserved positions tend to have higher average substitution scores.

The initial analysis of the structure-based MSA, including ordering the sequences by family and annotation, was performed in Jalview 2.11.4.1 (59). The structure-based MSA shown in Figure 3 and the BLOSUM62 average pairwise conservation plot shown in Fig. *S9* were produced by parsing the DALI-generated alignment using Biopython's AlignIO module (60) and visualized by matplotlib (61).

### DALI database search

The bacterial AT3 protein OafB (UniProt ID: A0A0H2WM30) was used as a query in the DALI search against the Human AlphaFold database. Strong matches to OafB were identified by a Z-score greater than 10.

### Maximum likelihood phylogenetic tree

The amino acid sequences of the known or predicted membrane components (trimmed to 10TMH helices or nine in the case of TraX, and seven in the case of DUF3623 and DUF3120) (Fig. S8) of the relevant proteins were aligned using MUSCLE 3.8 (62, 63). The maximum-likelihood phylogenetic tree was calculated using PhyML (64) using the Blosum62 substitution model and approximate likelihood ratio tests (aLRT) on the Galaxy platform (65). The resulting tree was visualised on Geneious Prime 2025.1.2 (https://www.geneious.com).

The tree was left unrooted, as no suitable outgroup could be identified. Although MBOAT was used as an outgroup in other analyses, it was not suitable for rooting the phylogenetic tree, since an ideal outgroup must be related yet sufficiently divergent, but MBOAT proteins are unrelated to TmAT family members. PSI-BLAST searches using the HGSNAT sequence returned only insignificant matches (E-value > 1), indicating unrelated sequences or hits corresponding to HGSNAT itself.

Hidden Markov Model (HMM) generation and searchThe following HMM’s were obtained from Pfam; HGSNAT (PF07768), GWT1 (PF06423), Cas1_AcylT (PF07779), AT3 (PF01757), TraX (PF05857), OpgC-C (PF10129), DUF418 (PF04235), DUF3120 (PF11318), DUF3623 (PF12291), and MBOAT (PF03062). Within Pfam, the HMM for DUF5009 (PF16401) was derived from a seed alignment containing only two sequences. To improve its reliability, we instead constructed a new HMM using the full alignment from Pfam and the hmmbuild command from the HMMER package (66). To represent the revised Pfam clan (TmAT), we generated a structure-based MSA using DALI and similarly built a custom HMM using hmmbuild (Fig. S12).

To identify significant protein matches to a given HMM, we used hmmsearch with the thresholds (--E 0.0001 --domE 0.0001 --incE 0.0001 --incdomE 0.0001) against the reference proteomes database. The number of proteins containing the domain was counted. If the aligned HMM region did not overlap with any known domain, it was classified as ‘unknown’. If it overlapped with a domain not discussed in this study, it was labeled ‘other’. Some domain hits included regions that overlapped with other domains not discussed in this study. In such cases, if one of the overlapping domains was HGSNAT, DUF5009, AT3, DUF418, OpgC-C, TraX, Cas1_AcylT, GWT1/PigW, DUF3623, DUF3120, or MBOAT, the region was classified as a hit for that domain. All non-zero hits were highlighted in bold (See Table S3).

The structure-based MSA generated from DALI (Fig. 3) was used to produce the stacked sequence logos shown in Fig. S13. For each input structure, DALI creates a sequence profile by identifying homologous sequences from UniProt. The TmAT HMM we generated was then compared against individual HMMs from the TmAT superfamily members (Fig. S12). The six conserved TmAT residues identified from the MSA are boxed (Fig. 3). Box positions were determined using the DALI stacked sequence logos (Fig. S13), which preserved alignment to the structural MSA, allowing residue mapping even when HMMs included insertions or deletions. All HMMs were aligned relative to the conserved Asp residue in the first TMH. The DUF418 HMM primarily captures only the C-terminal portion of TmAT superfamily proteins containing this domain while the DUF5009 does not fully capture the N-terminal portion. Due to the lack of an observable DxxR motif, the DUF418 HMM was aligned to the conserved Tyr in the ninth TMH.

## Data availability

All data is presented in this manuscript or could be reproduced using the sequence IDs provided in the data Table or Supplementary Tables.

## Supporting information

This article contains supporting information (2, 4, 8, 13, 14, 15, 16, 26, 56).

## Conflict of interest

The authors declare that they have no conflicts of interest with the contents of this article.
