# Supplementary material for: Structural unification of diverse transmembrane acyltransferases reveals a conserved fold for the transmembrane acyl transferase (TmAT) superfamily
Source: J Biol Chem. 2025 Aug 5;301(9):110546. doi: 10.1016/j.jbc.2025.110546 (PMC12423673; doi:10.1016/j.jbc.2025.110546)
Supplement: Supplementary Information [file mmc1.pdf]

Supporting Information

Structural unification of diverse membrane-bound acyltransferases reveals a conserved fold that defines the Transmembrane Acyl Transferase (TmAT) superfamily

Bethan E. Kinniment-Williams[1,2], Vytaute Jurgeleviciute[2,4], Daniel T. West[2,3], Reyme Herman[2,3], James N. Blaza[2,4], Marjan W. van der Woude[1,2], Gavin H. Thomas[2,3,*]

[1]Hull York Medical School, University of York, York, UK; [2]York Biomedical Research Institute, University of York, York, UK; [3]Department of Biology, University of York, York, UK & [4]York Structural Biology Laboratory, Department of Chemistry, University of York, York, UK.

[*]Corresponding author: gavin.thomas@york.ac.uk

Fig. S1-S15

Table S1-S7

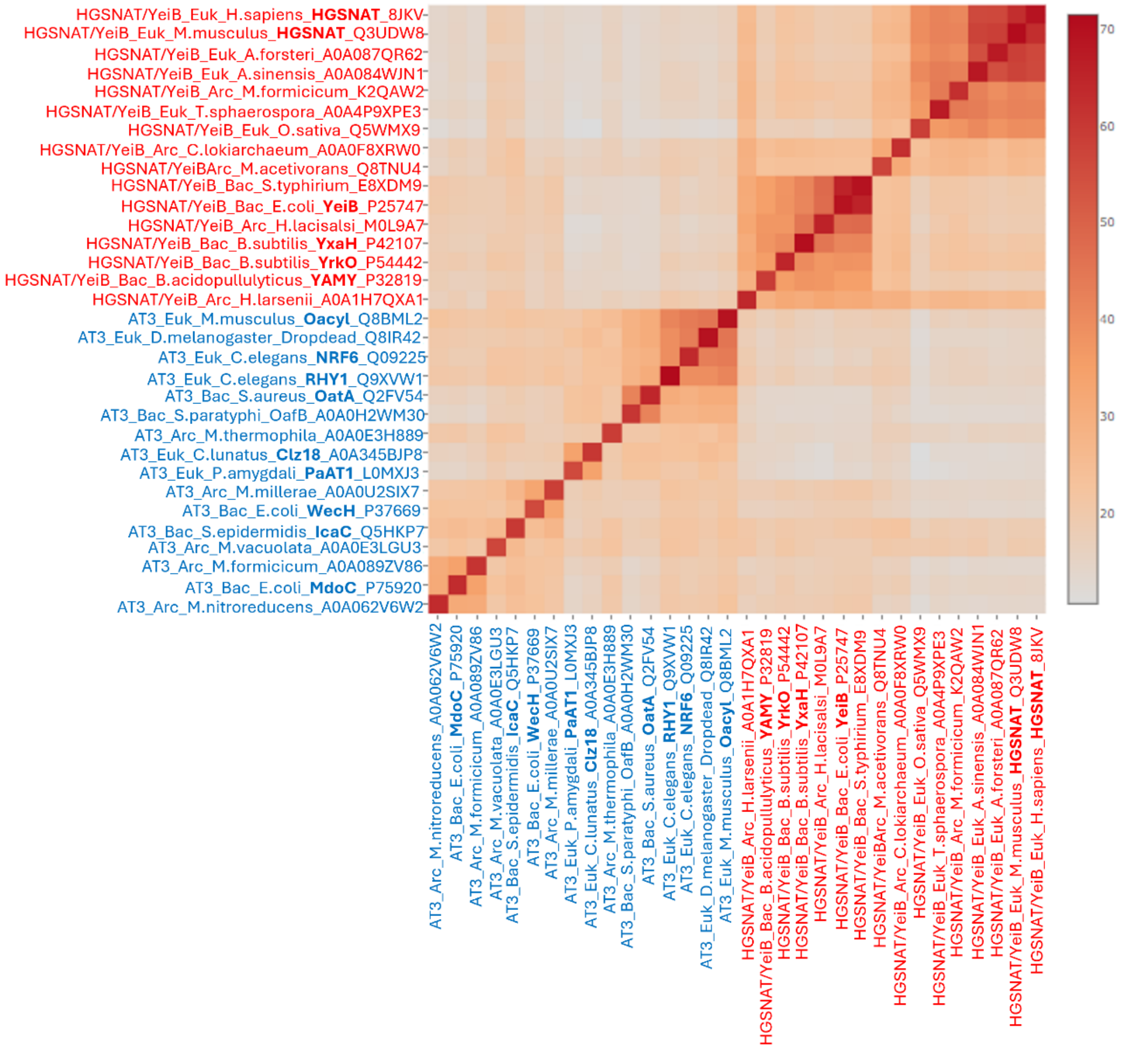

Fig. S1. Heat map of Z-scores of a DALI all against all alignment of select members of the two TDBC-defined families (AT3 and HGSNAT/YeiB) within the TmAT superfamily. Z scores of 16 members with representative members from archaea (Arc), bacteria (Bac) and eukaryota (Euk) are displayed. AT3 members are coloured in blue, while HGSNAT/YeiB members are coloured in red. The Z-score between two proteins is useful in determining homology: a Z-score below 2 suggests no significant similarity, while a score between 8 and 20 indicates a medium likelihood of homology and over 20, a high likelihood (56). As expected, AT3 proteins show high Z-scores (13.7-45.3) when compared to each other, and HGSNAT/YeiB family proteins also display high Z-scores within their group (16.0-62.6). The protein members of the AT3 and HGSNAT/YeiB families appear to be at least distantly related, with all Z-scores being 10.5 or higher, confirming significant structural similarity across the TCDB-defined TmAT superfamily.

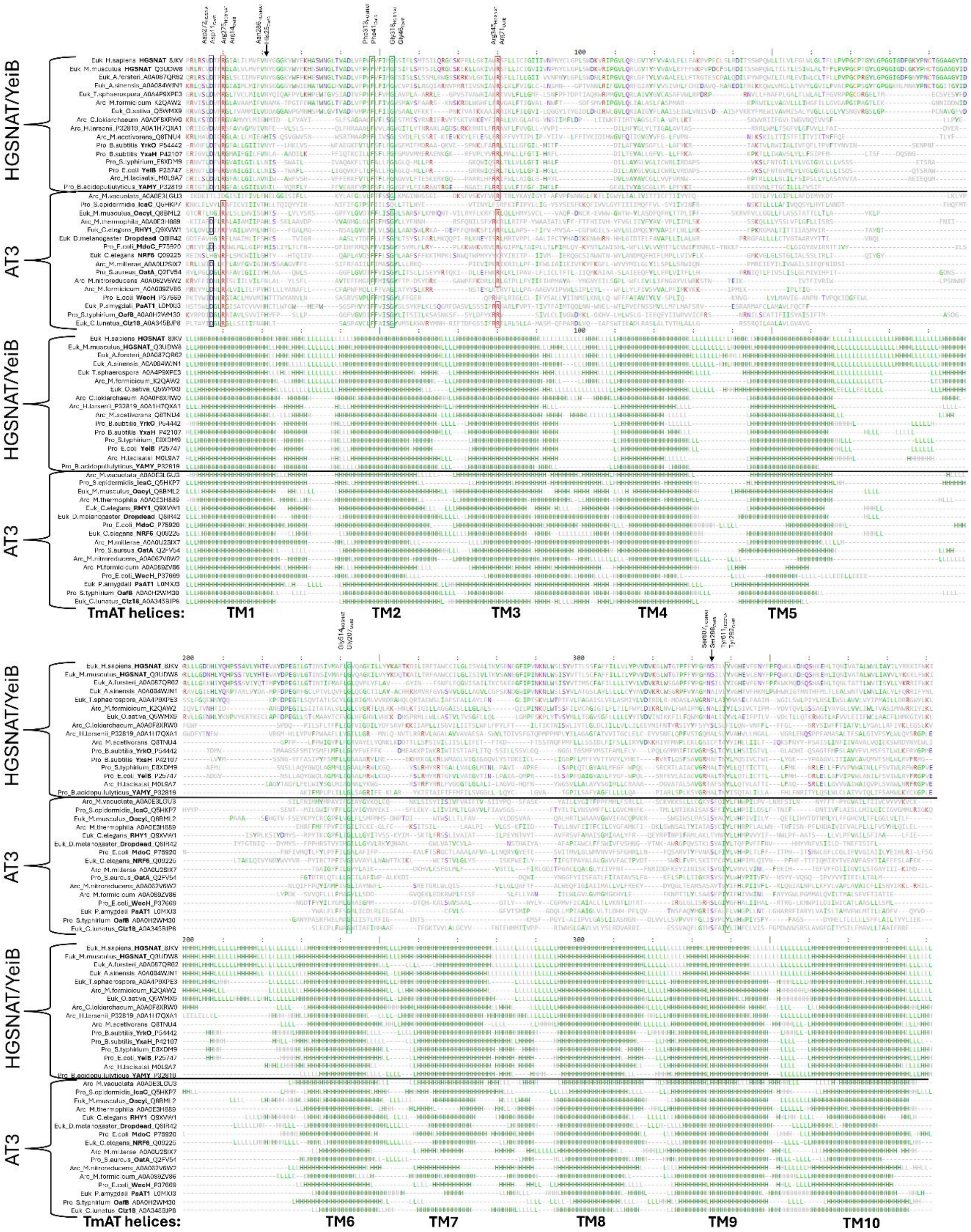

Fig. S2. Structure-based sequence alignment of select AT3 and HGSNAT/YeiB family members. The alignments were produced using DALI and HGSNAT (PDB: 8JKV) as the reference structure. Regions lacking any sequence/structural similarity are not expanded, so only structurally equivalent positions to HGSNAT (PDB: 8JKV) are shown. The most common amino acid at any given position is coloured according to aromaticity (dark green)(Phe, His, Trp, Tyr), positive charge (red) (Arg, Lys), negative charge (blue) (Glu, Asp), polar uncharged side chains (purple) (Ser, Thr, Gln, Asn), hydrophobic side chains that are not aromatic (Gly, Leu, Ile, Val, Pro, Met, Ala), and sulfur-containing amino acid (orange) (Cys). Boxed residues are conserved or exhibit conservative substitutions (Phe↔Tyr,

Arg↔Lys, Asp↔Glu) across the alignment. Additional partially conserved residues discussed in the text are marked with an arrow. Protein secondary structure is annotated as H - Helix, L - Coil, and E -Sheet.

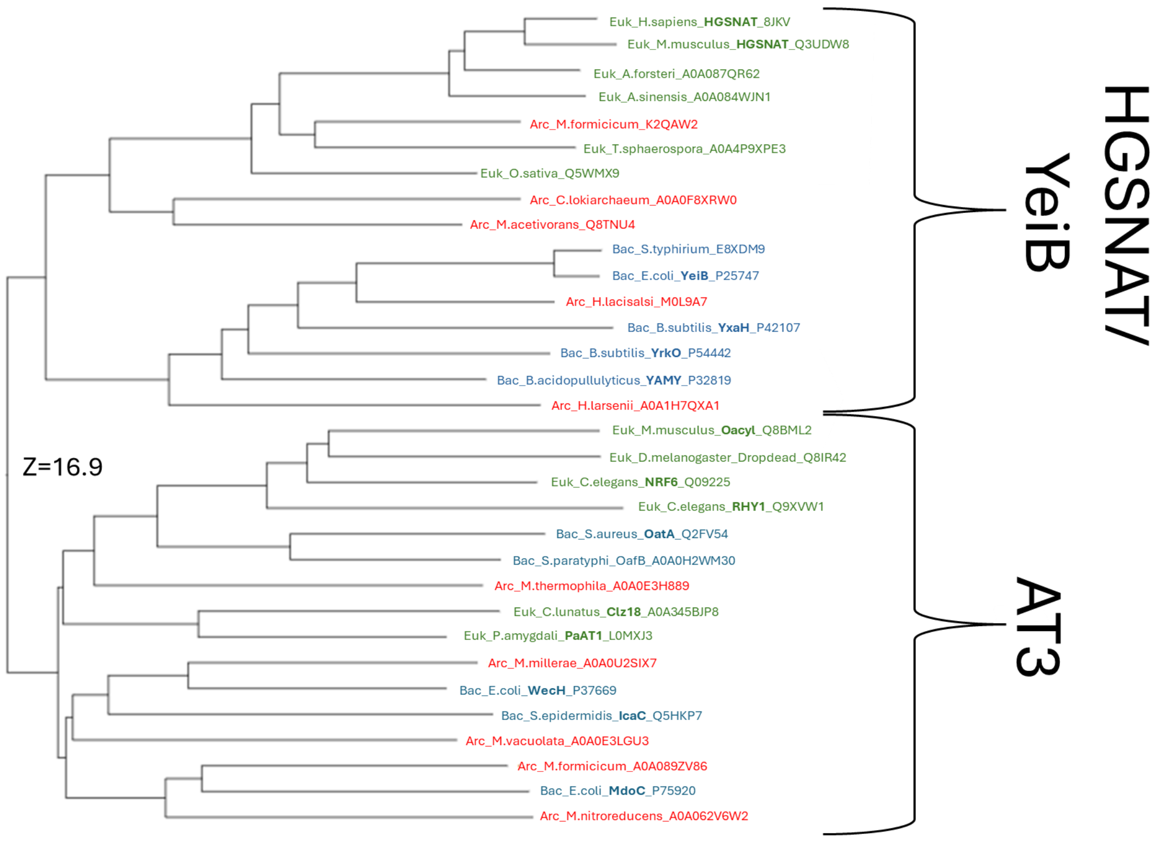

Fig. S3. Structural dendrogram of select AT3 and HGSNAT/YeiB family members. The structural dendrogram shows that the standalone AT3 and HGSNAT/YeiB families form two separate clades. Representative members from archaea (Arc), bacteria (Bac) and eukaryota (Euk) are coloured in red, blue and green respectively.

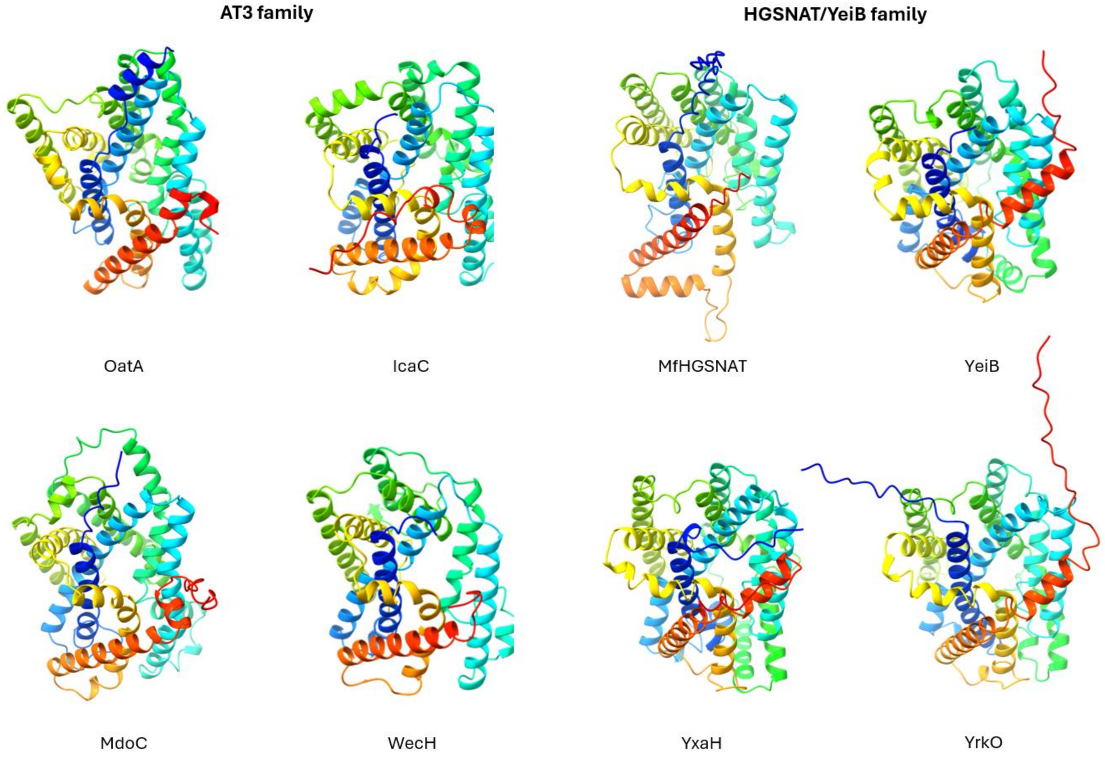

Fig. S4. Conservation of a core 10-TMH TmAT protein fold within the two TDBC-defined families. A cytosolic view of representative members of the AT3 and HGSNAT/YeiB families. Any additional domains and helices have been removed to illustrate the TmAT fold. Proteins are all coloured with rainbow colouring (N-terminus to C-terminus). All structures are Alphafold models. AT3 family representatives are OatA (Uniprot ID: Q2FV54), IcaC (Uniprot ID: Q5HKP7), MdoC (Uniprot ID: P75920) and WecH (Uniprot ID: P37669). HGSNAT/YeiB family representative members are MfHGSNAT (Uniprot ID: K2QAW2), YeiB (Uniprot ID: P254747), YxaH (Uniprot ID: P42107) and YrkO (Uniprot ID: P54442).

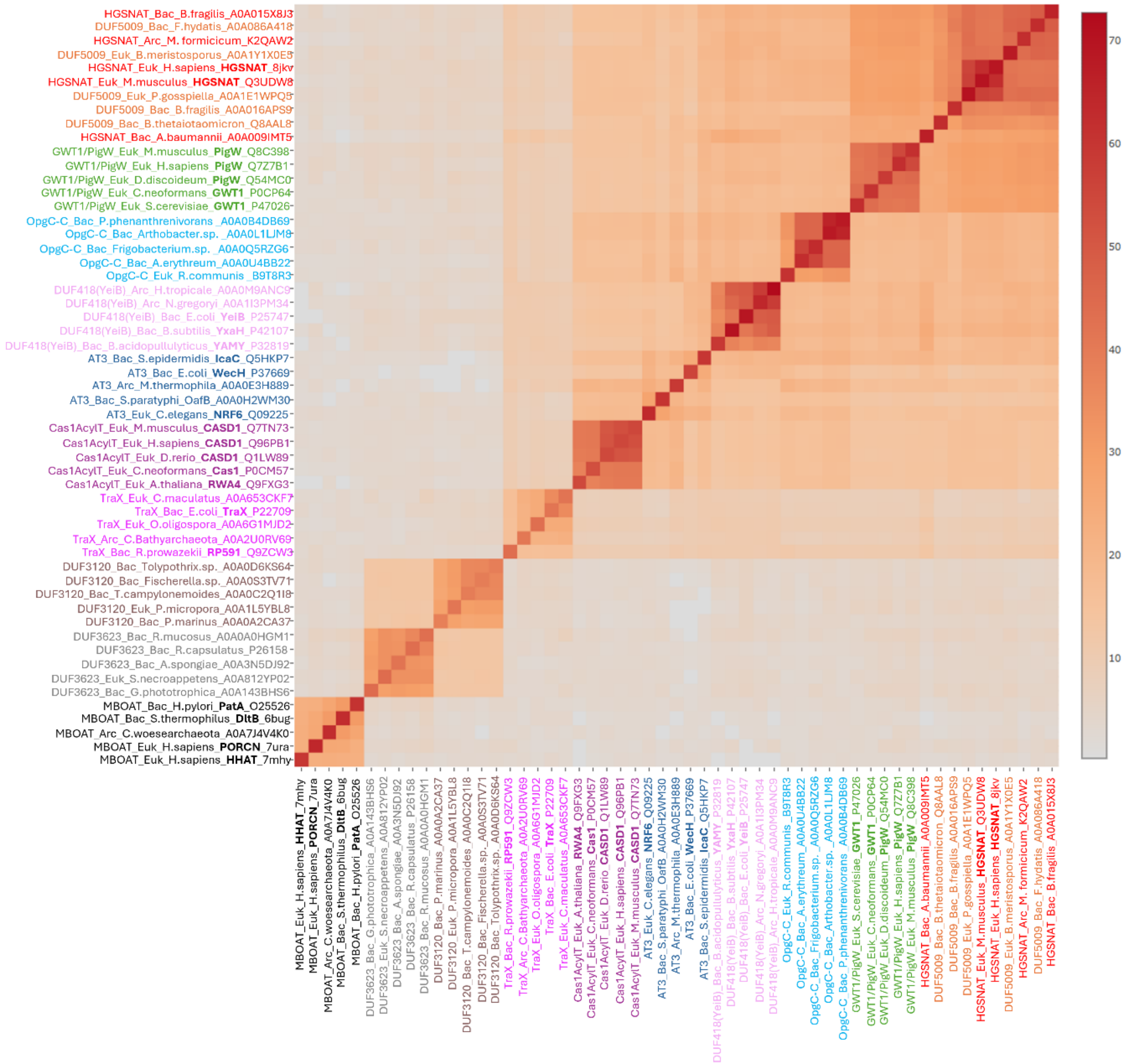

Fig. S5. Heat map of Z-scores of a DALI all against all alignment of select members of the Pfam Acyl_transf_3 Clan (CL0316), GWT1/PIG-W family and MBOAT superfamily. Z scores of the 10 families and 1 superfamily investigated with 5 representative members in each including members from archaea (Arc), bacteria (Bac) and eukaryota (Euk) are displayed. Members are coloured according to family - AT3 in dark blue, HGSNAT/YeiB in red, DUF5009 in orange, GWT1/PIG-W in green, DUF418 in light pink, OpgC-C in light blue, Cas1_AcylT in maroon, TraX in purple, DUF3120 in brown, DUF3623 in grey and MBOAT in black. MBOAT, when compared with all other proteins (excluding DUF3623, DUF3120, and itself), shows a Z-score range of 0.1–5.4, as expected for an outgroup. In contrast, the Z-score range between the Pfam Acyl_transf_3 Clan (CL0316) members plus the GWT1/PIG-W family members, is 11.9–48.6, clearly indicating these are members of the TmAT superfamily. For TraX, the Z-score range with the Pfam Acyl_transf_3 Clan (CL0316) plus the GWT1/PIG-W family is 6.1–13.1, suggesting it is a borderline member of the TmAT superfamily.

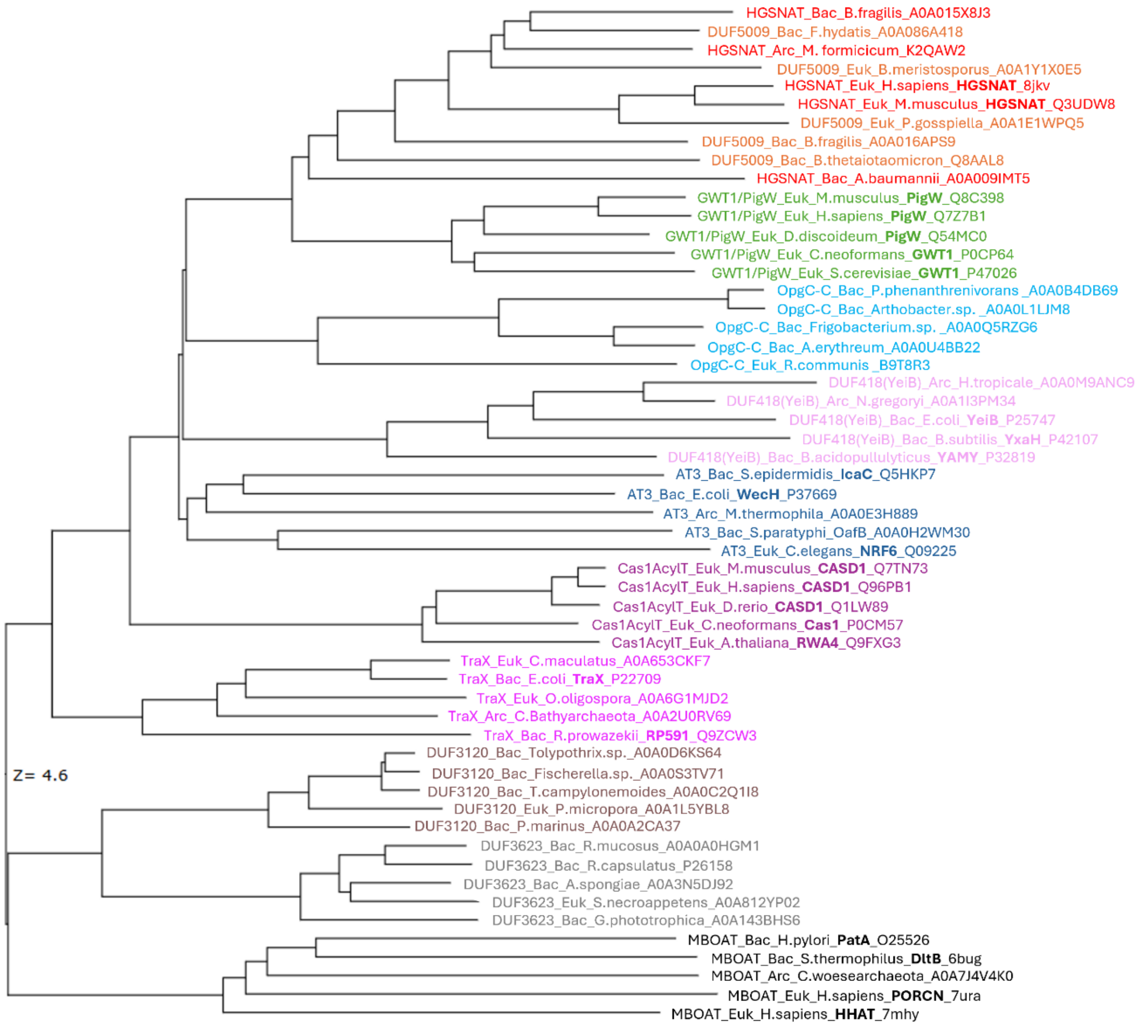

Fig. S6. Structural dendrogram of select members of the Pfam Acyl_transf_3 Clan (CL0316), GWT1/PIG-W family and MBOAT superfamily. Each group contains 5 representative proteins including members from archaea (Arc), bacteria (Bac) and eukaryota (Euk) where possible. The structural dendrogram shows that AT3, HGSNAT/YeiB, DUF5009, GWT1/PIG-W, DUF418, OpgC-C, Cas1_AcylT and TraX families are structurally related. It also highlights that the MBOAT superfamily is not structurally related to the other investigated families. Members are coloured according to family - AT3 in dark blue, HGSNAT/YeiB in red, DUF5009 in orange, GWT1/PIG-W in green, DUF418 in light pink, OpgC-C in light blue, Cas1_AcylT in maroon, TraX in purple, DUF3120 in brown, DUF3623 in grey and MBOAT in black.

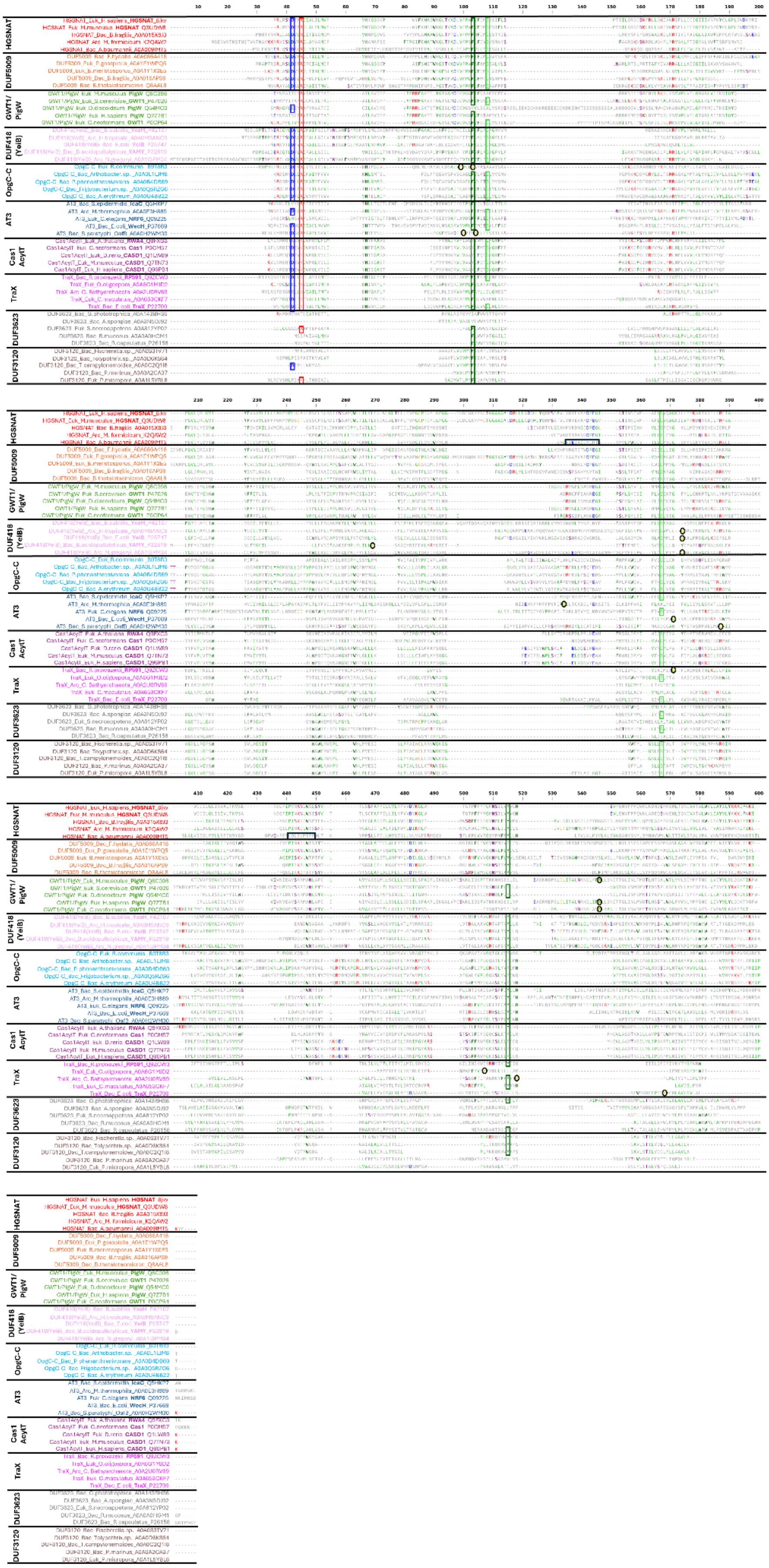
HGSNAT
DUF5009
GWT1/ PigW
DUF418 (YeiB)
OpgC-C
AT3
Cas1 AcylT
TraX
DUF3623
DUF3120

Fig. S7 Multiple sequence alignment of select members of the Pfam Acyl_transf_3 Clan (CL0316), GWT1/PIG-W family which was used to generate the ML phylogenetic tree in Fig S8. The most common amino acid at any given position is coloured according to aromaticity (dark green) (Phe, His, Trp, Tyr), positive charge (red) (Arg, Lys), negative charge (blue) (Glu, Asp), polar uncharged side chains (purple) (Ser, Thr, Gln, Asn), hydrophobic side chains that are not aromatic (Gly, Leu, Ile, Val, Pro, Met, Ala), and sulfur containing amino acid (orange) (Cys). Boxed residues are those that are conserved, or contain conservative substitutions (Phe↔Tyr, Arg↔Lys, Asp↔Glu), across the alignment. The six key conserved residues highlighted in Fig. 3 are also boxed here. Yellow-filled circles mark positions where the structural MSA and this sequence-based MSA differ. The two highlighted rectangles indicate major sequence and structural differences between A0A009IMT5 and the other HGSNAT family members, explaining A0A009IMT5's distant placement from other HGSNAT members in the ML tree in ***Fig. S8***.

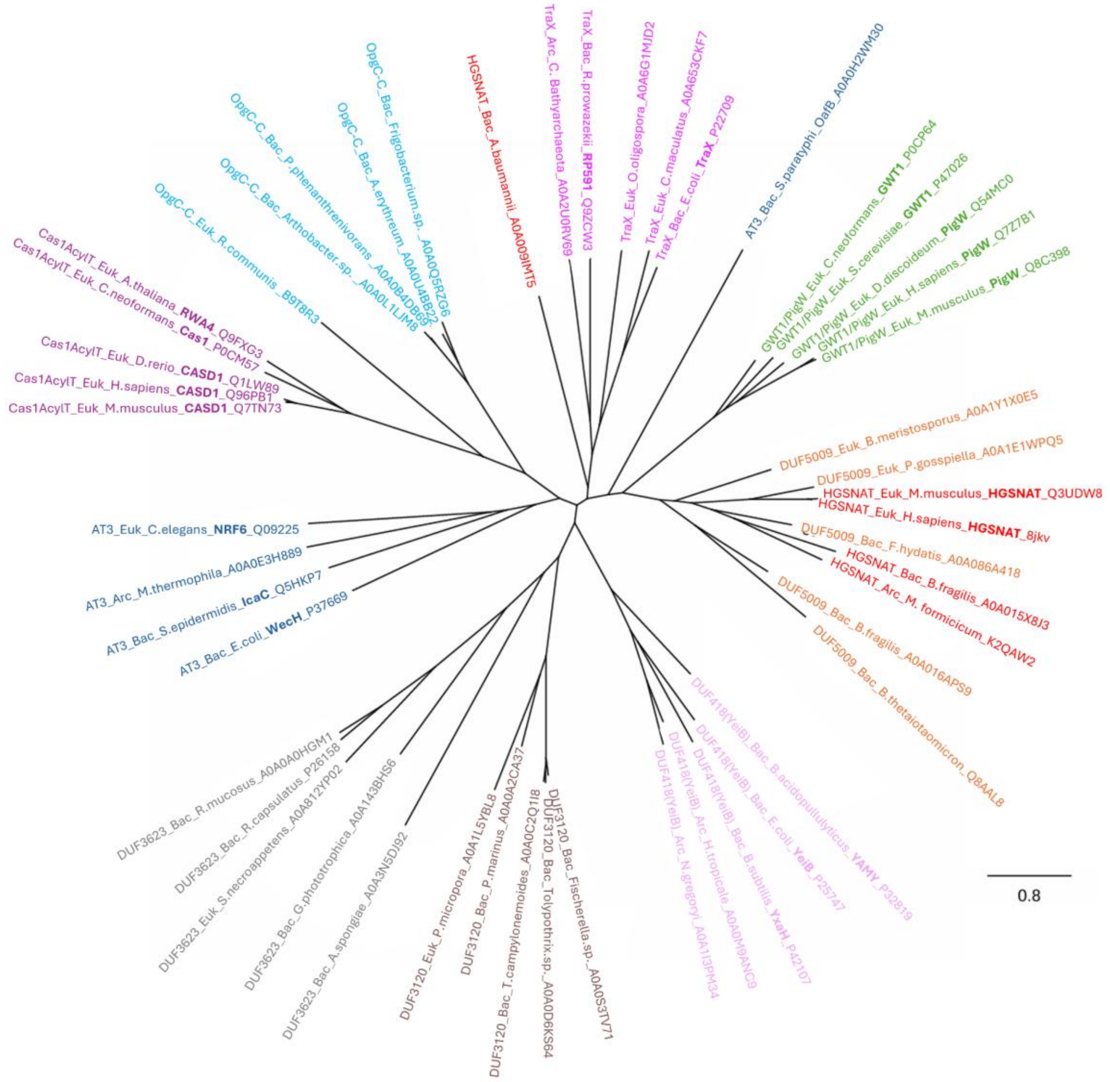

Fig. S8 . An unrooted maximum likelihood phylogenetic tree of select members of the Pfam Acyl_transf_3 Clan (CL0316), GWT1/PIG-W family. Families are grouped by colour. Refining the TmAT superfamily remains challenging when solely relying on sequence alignments, given the low sequence similarity between these proteins.. This demonstrates the limitations of relying solely on sequence alignments, as opposed to structural analysis, for protein classification.

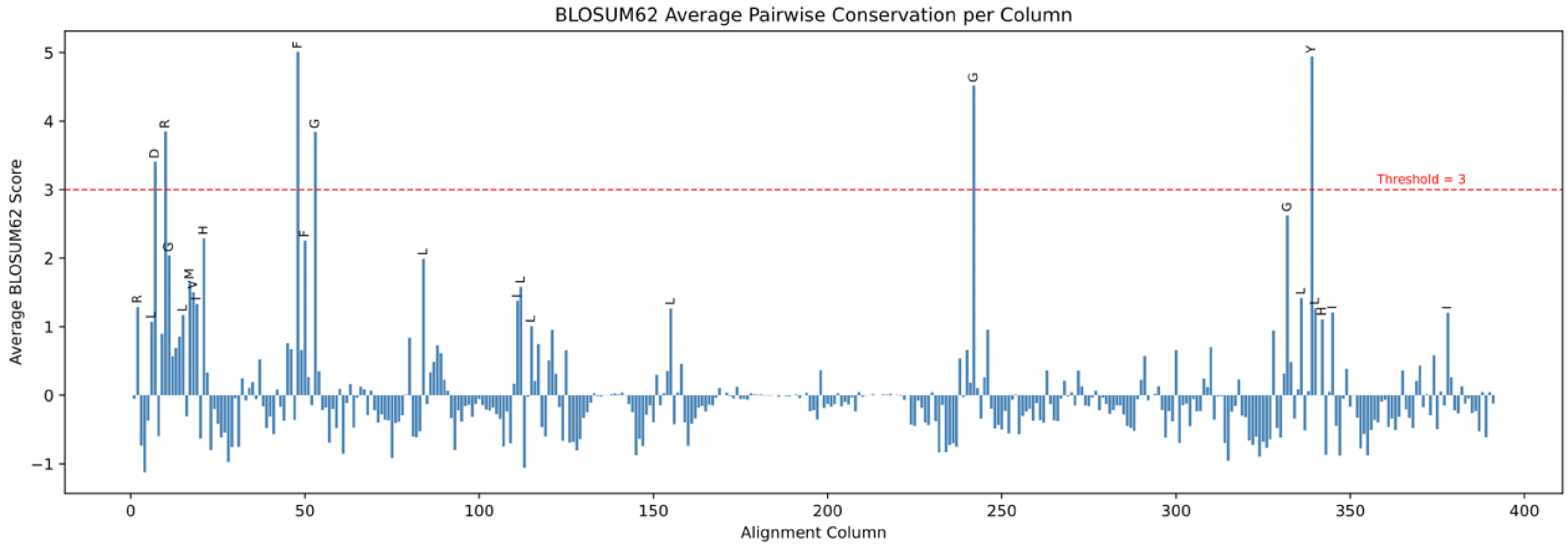

Fig S9. Bar chart of average pairwise BLOSUM62 conservation scores per alignment column for the structural multiple sequence alignment in Fig.3. Each bar represents the normalised average BLOSUM62 score for all residue pairs in that column, accounting for gaps by dividing by the total number of sequences in the alignment. The most frequent (i.e. most conserved) amino acid is displayed for every bar with a >1 average BLOSUM62 score. Higher bars reflect greater sequence conservation. A red dashed line at a score of 3 highlights the threshold for identifying the six highly conserved residues across the TmAT superfamily.

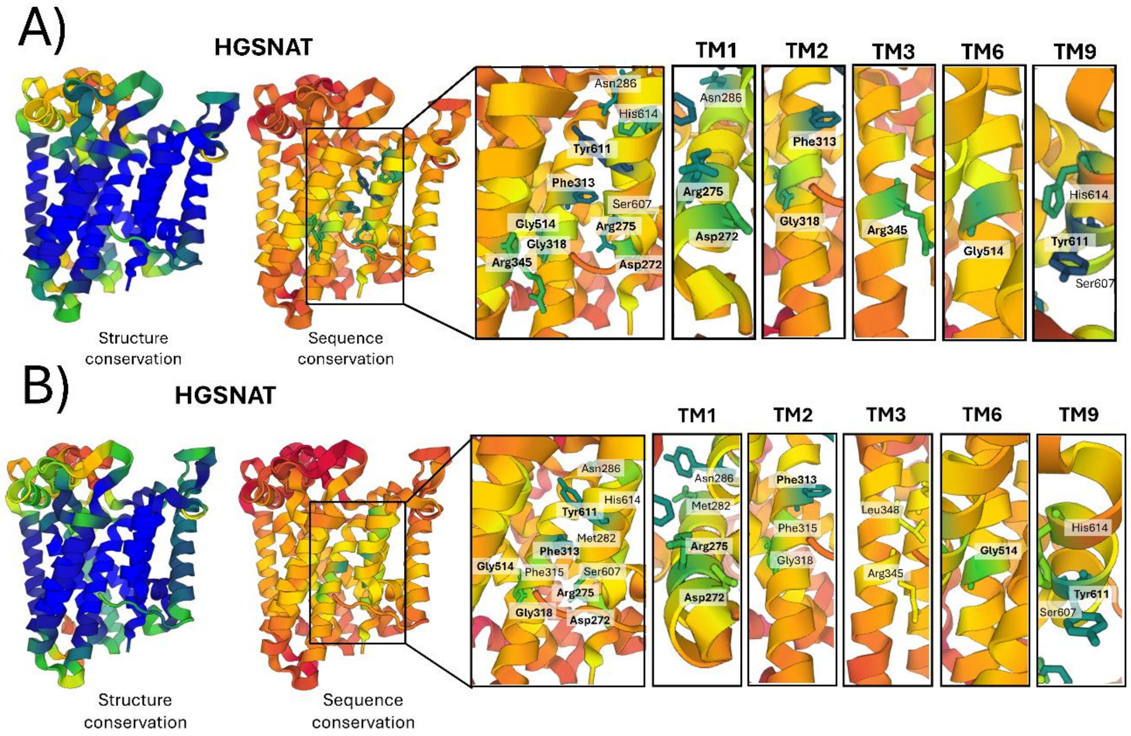

Fig. S10. Protein structure and amino acid position conservation between select members containing a TmAT domain. A) Conserved amino acid positions across the TCDB-defined TmAT superfamily (HGSNAT/YeiB and AT3 family members that are truncated to only include the core 10 TM TmAT domain) overlaid on the TmAT domain of human HGSNAT (PDB: 8JKV) structure generated using DALI. B) Conserved amino acid positions across the TmAT superfamily (HGSNAT, DUF418/YeiB, OpgC-C, AT3, Cas1_AcylT, PigW/GWT1, and TraX) (cut to 10TM) overlaid on the human HGSNAT (PDB: 8JKV) structure generated using DALI. The left models show structural conservation while the right shows sequence conservation. A colour gradient from blue to red is used to indicate conservation with blue indicating the most conserved features and red indicating the least conserved features. Panels on the right-hand side highlight the conservation of residues in TMH 1-3, 6 and 9 (using TmAT helical numbering). Highly conserved residues are highlighted in bold.

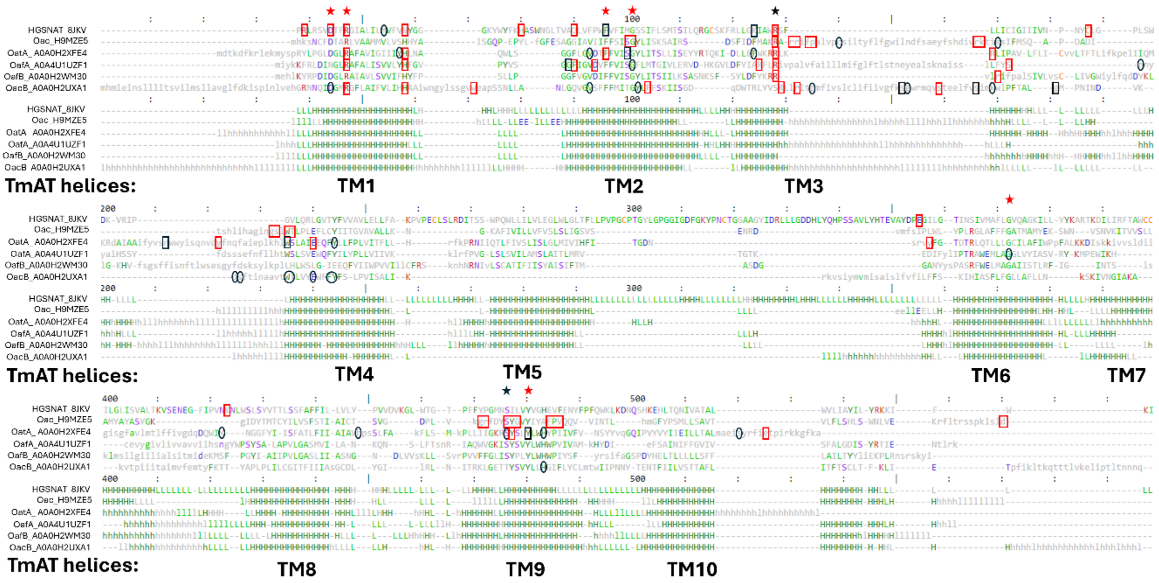

Fig. S11.Structure-based sequence alignment highlighting amino acid residues in HGSNAT and select AT3 members that have been functionally analysed by mutagenesis. HGSNAT (PDB ID: 8JKV), Oac (Uniprot ID: H9MZE5), OatA (Uniprot ID: A0A0H2XFE4), OafA (Uniprot ID: A0A4U1UZF1), OafB (Uniprot ID: A0A0H2WM30), and OacB (Uniprot ID: A0A0H2UXA1) were truncated to include only their 10TM TmAT domains. Mutated residues, highlighted by black boxes, have been identified as affecting protein function. A red box marks residues deemed critical to protein activity, either based on supporting literature (2, 4, 8, 13-16) or when mutations result in at least a 50% reduction in activity. Residues that are circled in black were also mutated but were found to be non-critical. In cases where multiple residues are boxed together, it indicates that they were mutated simultaneously. The 6 residues we identify as being conserved across the TmAT superfamily are marked with a red star above. The additional partially conserved residue, Ser607$_{HGSNAT}$ and Arg345$_{HGSNAT}$, that is discussed in the text is marked with a black star. Protein secondary structure is annotated as H - Helix, L - Coil, and E -Sheet.

Figure is in a separate .pdf document

Fig. S12. HMMs for each member of the TmAT superfamily aligned to the conserved Asp residue within the DxxR motif. The six key conserved TmAT residues are boxed where identifiable. The location of the six key residues were identified from using Fig S12. An asterisk (*) indicates a boxed position where alignment is uncertain due to poor sequence conservation. DUF5009 and DUF418 HMMs only capture the start and end regions of the corresponding DUF5009- and DUF418-containing proteins, respectively.

Figure is in a separate .pdf document

Fig. S13. Stacked sequence logos generated by DALI for each of the 55 TmAT input structures. For each structure, DALI identified homologous sequences from UniProt and constructed a sequence profile. Members of different TmAT domain families are color coded. The six key conserved TmAT residues are highlighted with boxe.

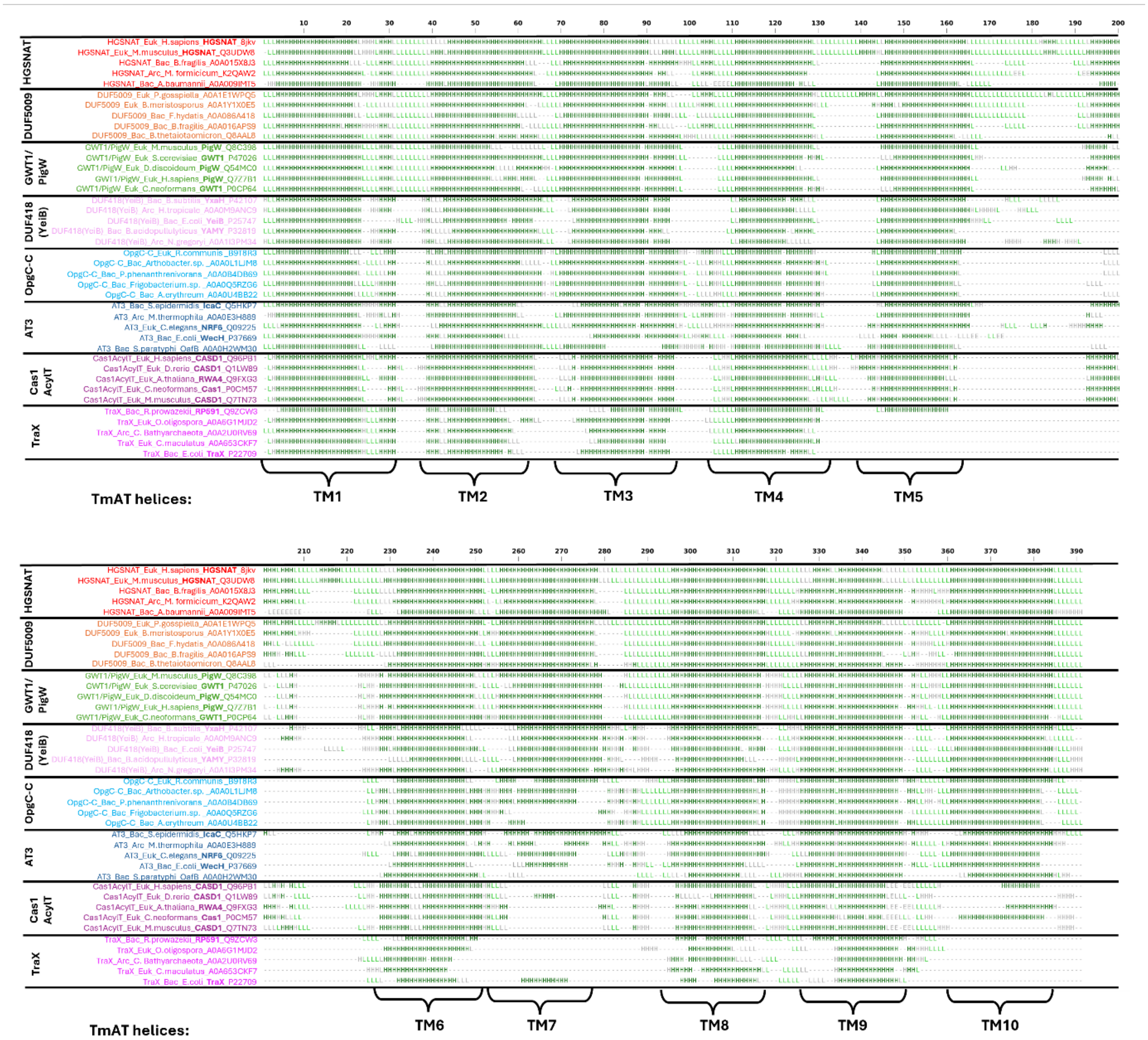

Fig. S14. Secondary structure alignment of the TmAT superfamily members. Secondary structure alignment (corresponding to **Fig.3**) compared to HGSNAT 8JKV) produced in DALI showing diverse representatives of Pfam Acyl_transf_3 Clan (CL0316) members containing the TmAT domain and the additional PigW/GWT1 family found by a DALI search. Regions with no structural/sequence homology are not expanded, so only structurally equivalent positions to HGSNAT (8JKV) are shown. It is important to note that some of the proteins appear to not have a TM5 or TM7, but this is due to poor structural alignment with other TmAT members and not because they lack a TM5 or 7. Protein secondary structure is annotated as H - Helix, L - Coil, and E -Sheet.

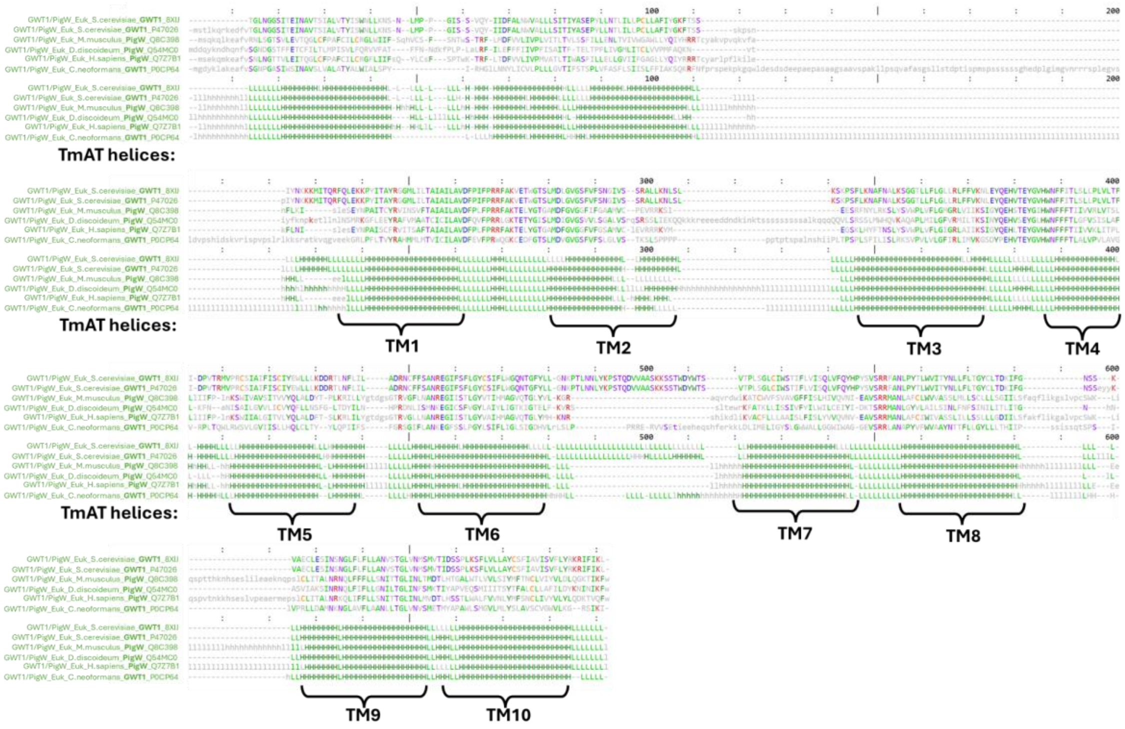

Fig. S15. Structure-based multiple sequence alignment of GWT1/PigW proteins used in the analysis compared to the recently solved structure of GWT1 (PDB ID: 8XIJ). The alignments were produced using DALI and GWT1 (PDB: 8XIJ) as the reference structure. This alignment demonstrates that the sequence and structure of the resolved GWT1 closely resemble the AlphaFold GWT1/PigW structures, particularly for the GWT1 protein from *Saccharomyces cerevisiae* (Uniprot ID: P47026). Regions lacking any sequence/structural similarity are not expanded, so only structurally equivalent positions to GWT1 (PDB: 8XIJ) are shown. The most common amino acid at any given position is coloured according to aromaticity (dark green)(Phe, His, Trp, Tyr), positive charge (red)(Arg, Lys), negative charge (blue)(Glu, Asp), polar uncharged side chains (purple) (Ser, Thr, Gln, Asn), hydrophobic side chains that are not aromatic (Gly, Leu, Ile, Val, Pro, Met, Ala), and sulfur-containing amino acid (orange) (Cys). Protein secondary structure is annotated as H - Helix, L - Coil, and E - Sheet.

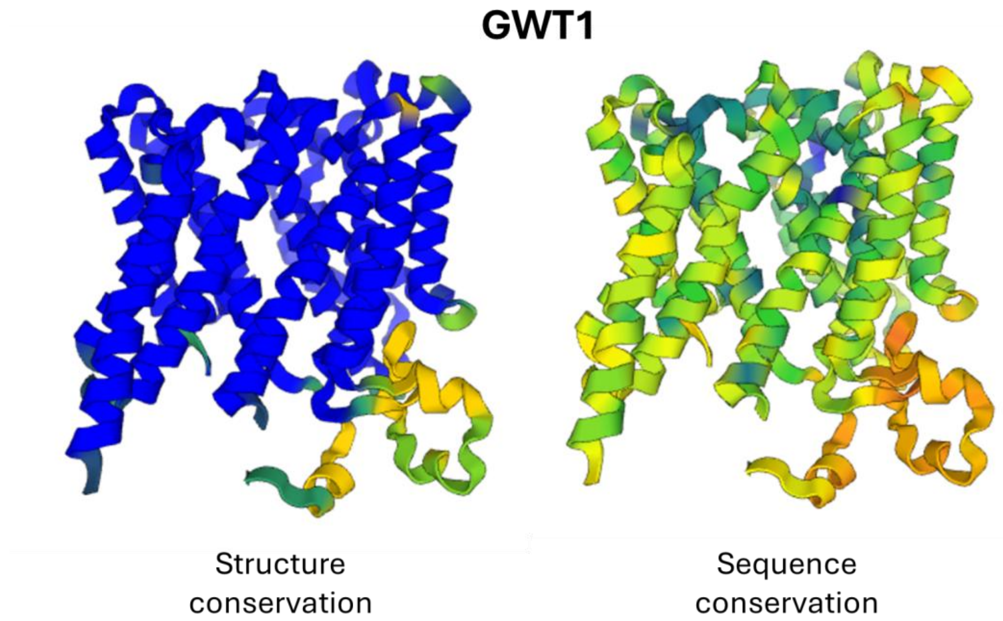

Fig. S16. Protein structure and amino acid position conservation between GWT1/PigW proteins used in the analysis and the recently solved structure of GWT1 (PDB ID: 8XIJ) Conserved amino acid positions across the GWT1 superfamily (Uniprot IDs: P47026, Q8C398, Q54MC0, Q7Z7B1, P0CP64) overlaid on the GWT1 (PDB: 8XIJ) structure generated using DALI. The left models show structural conservation while the right shows sequence conservation. A colour gradient from blue to red is used to indicate conservation with blue indicating the most conserved features and red indicating the least conserved features. The structural and sequence conservation demonstrate that the solved structure of GWT1 closely resembles the AlphaFold GWT1/PigW structures used in this analysis.

Table S1. **The 32 proteins analysed within the TCDB-defined TmAT superfamily, along with the corresponding residues to which each structure was truncated.**

Protein	Residues *
HGSNAT/YeiB_Euk_H.sapiens_HGSNAT_8jkv	266-663
HGSNAT/YeiB_Euk_T.sphaerospora_A0A4P9XPE3	68-472
HGSNAT/YeiB_Euk_A.sinensis_A0A084WJN1	188-577
HGSNAT/YeiB_Euk_O.sativa_Q5WMX9	1-491 (uncut)
HGSNAT/YeiB_Euk_A.forsteri_A0A087QR62	159-551
HGSNAT/YeiB_Euk_M.musculus_HGSNAT_Q3UDW8	257-656
HGSNAT/YeiB_Bac_E.coli_YeiB_P25747	1-385 (uncut)
HGSNAT/YeiB_Bac_B.subtilis_YrkO_P54442	1-405 (uncut)
HGSNAT/YeiB_Bac_B.subtilis_YxaH_P42107	1-402 (uncut)
HGSNAT/YeiB_Bac_S.typhirium_E8XDM9	1-386 (uncut)
HGSNAT/YeiB_Bac_B.acidopullulyticus_YAMY_P32819	1-346 (uncut)
HGSNAT/YeiB_Arc_H.larsenii_A0A1H7QXA1	1-364 (uncut)
HGSNAT/YeiB_Arc_C.lokiarchaeum_A0A0F8XRW0	1-373 (uncut)
HGSNAT/YeiB_Arc_M.acetivorans_Q8TNU4	1-353 (uncut)
HGSNAT/YeiB_Arc_H.lacisalsi_M0L9A7	1-432 (uncut)
HGSNAT/YeiB_Arc_M.formicicum_K2QAW2	1-382 (uncut)
AT3_Euk_D.melanogaster_Dropdead_Q8IR42	382-786
AT3_Euk_M.musculus_Oacyl_Q8BML2	266-685
AT3_Euk_P.amygdali_PaAT1_L0MXJ3	1-497 (uncut)
AT3_Euk_C.lunatus_Clz18_A0A345BJP8	1-469 (uncut)
AT3_Euk_C.elegans_NRF6_Q09225	372-822
AT3_Euk_C.elegans_RHY1_Q9XVW1	87-502
AT3_Bac_E.coli_WecH_P37669	1-331 (uncut)
AT3_Bac_S.epidermidis_IcaC_Q5HKP7	1-355 (uncut)
AT3_Bac_S.aureus_OatA_Q2FV54	1-368 (uncut)
AT3_Bac_E.coli_MdoC_P75920	1-385 (uncut)
AT3_Bac_S.paratyphi_OafB_A0A0H2WM30	1-343
AT3_Arc_M.formicicum_A0A089ZV86	1-364 (uncut)

AT3_Arc_M.nitroreducens_A0A062V6W2	1-379 (uncut)
AT3_Arc_M.millerae_A0A0U2SIX7	1-349 (uncut)
AT3_Arc_M.vacuolata_A0A0E3LGU3	1-245 (uncut)
AT3_Arc_M.thermophila_A0A0E3H889	1-431 (uncut)

*These residues are inclusive. If a protein already contained 10TMH and was not truncated, it is marked as (uncut).

Table S2. DALI structural alignment statistics of AT3 and HGSNAT/YeiB family members when compared to HGSNAT (PDB ID: 8JKV).

Protein	Z score	RMSD	Aligned residues	Total no. of residues	Sequence ID %
HGSNAT/YeiB_Euk_M.musculus_HGSNAT_Q3UDW8	62.6	0.8	391	400	88
HGSNAT/YeiB_Euk_A.forsteri_A0A087QR62	56.3	1.4	383	393	70
HGSNAT/YeiB_Euk_A.sinensis_A0A084WJN1	55.9	1.3	384	390	45
HGSNAT/YeiB_Euk_T.sphaerospora_A0A4P9XPE3	41.9	2.4	348	405	27
HGSNAT/YeiB_Arc_M.formicicum_K2QAW2	40.7	2.1	335	382	29
HGSNAT/YeiB_Euk_O.sativa_Q5WMX9	39.2	2.0	364	491	30
HGSNAT/YeiB_Arc_C.lokiarchaeum_A0A0F8XRW0	26.2	3.1	301	373	16
HGSNAT/YeiB_Arc_H.larsenii_A0A1H7QXA1	25.2	4.0	300	364	15
HGSNAT/YeiB_Arc_M.acetivorans_Q8TNU4	25.1	3.4	298	353	19
HGSNAT/YeiB_Bac_B.subtilis_YrkO_P54442	22.0	3.7	291	405	12
HGSNAT/YeiB_Bac_B.subtilis_YxaH_P42107	20.9	3.9	282	402	15
HGSNAT/YeiB_Bac_S.typhirium_E8XDM9	19.3	4.0	278	386	16
HGSNAT/YeiB_Bac_E.coli_YeiB_P25747	19.3	3.9	280	385	17
AT3_Arc_M.vacuolata_A0A0E3LGU3	18.9	4.1	280	345	14
HGSNAT/YeiB_Arc_H.lacisalsi_M0L9A7	18.8	4.3	283	432	17
HGSNAT/YeiB_Bac_B.acidopullulyticus_YAMY_P32819	18.3	3.8	263	346	16
AT3_Bac_S.epidermidis_IcaC_Q5HKP7	18.2	4.7	286	355	10
AT3_Euk_M.musculus_Oacyl_Q8BML2	16.3	4.7	291	420	10
AT3_Euk_C.elegans_RHY1_Q9XVW1	15.7	5.0	285	416	13
AT3_Arc_M.thermophila_A0A0E3H889	15.6	4.4	272	431	14
AT3_Euk_D.melanogaster_Dropdead_Q8IR42	15.1	4.8	276	405	13
AT3_Bac_E.coli_MdoC_P75920	14.9	4.9	289	385	11
AT3_Euk_C.elegans_NRF6_Q09225	14.9	4.7	293	451	13
AT3_Arc_M.millerae_A0A0U2SIX7	14.5	4.7	261	349	11
AT3_Bac_S.aureus_OatA_Q2FV54	14.0	4.7	250	368	16
AT3_Arc_M.nitroreducens_A0A062V6W2	13.8	4.5	271	379	13
AT3_Bac_E.coli_WecH_P37669	13.5	5.7	250	331	13
AT3_Arc_M.formicicum_A0A089ZV86	13.4	4.4	248	364	10
AT3_Euk_P.amygdali_PaAT1_L0MXJ3	13.3	4.6	266	497	15
AT3_Bac_S.paratyphi_OafB_A0A0H2WM30	13.2	5.0	250	343	12
AT3_Euk_C.lunatus_Clz18_A0A345BJP8	13.2	4.5	258	469	12

Table S3. Number of protein hits with the following domains when searching with protein HMMs in hmmsearch (E-value < 1×10^{-4}). Dark orange shading highlights the HGSNAT, DUF5009, AT3, DUF418, and OpgC-C families, which show many reciprocal hits (except for no hits between DUF5009 and OpgC-C for DUF418, and none from DUF5009 to OpgC-C). Lighter orange shading highlights TraX, with three hits to AT3 domains, indicating borderline similarity. No hits were found between TmAT superfamily members and Cas1_AcylT or GWT1. MBOAT (outgroup) shows no similarity to any TmAT members. DUF3623 and DUF3120 also have no hits, supporting their exclusion from the TmAT superfamily. Non-zero numbers are in bold. In the "Other" category, the name of the protein is given in brackets. In "Unknown," no domain was assigned to the protein hit.

Protein Hits by Domain (hmmsearch, E < 1×10^{-4})	Protein HMM											
	HGSNAT	DUF5009 (full)	AT3	DUF418	OpgC-C	TraX	Cas1_ AcylT	GWT1	DUF3120	DUF3623	MBOAT	TmAT
HGSNAT	**3386**	**3144**	**567**	**69**	**5**	0	0	0	0	0	0	**4910**
DUF5009	**6**	**548**	**1**	0	0	0	0	0	0	0	0	**496**
AT3	**117**	**18**	**40622**	**15**	**24**	0	0	0	0	0	0	**6863**
DUF418	**135**	**10**	**162**	**4156**	0	0	0	0	0	0	0	**2981**
OpgC-C	**8**	**1**	**282**	0	**1389**	0	0	0	0	0	0	**1051**
TraX	0	0	**3**	0	0	**1303**	0	0	0	0	0	**65**
Cas1_AcylT	0	0	0	0	0	0	**3291**	0	0	0	0	**3058**
GWT1	0	0	0	0	0	0	0	**3067**	0	0	0	**2151**
DUF3120	0	0	0	0	0	0	0	0	**190**	0	0	0
DUF3623	0	0	0	0	0	0	0	0	0	**387**	0	0
MBOAT	0	0	0	0	0	0	0	0	0	0	**24861**	0
HGSNAT/ AT3	0	0	**1**	0	0	0	0	0	0	0	0	0
HGSNAT/ DUF5009	**1**	**10**	0	0	0	0	0	0	0	0	0	**138**
HGSNAT/ DUF418	0	0	**5**	**2**	0	0	0	0	0	0	0	**27**
AT3/ DUF418	**11**	0	0	0	0	0	0	0	0	0	0	**40**
AT3/DUF418/	0	0	0	0	0	0	0	0	0	0	0	**1**

**OpgC-C**												
**DUF418/ OpgC-C**	0	0	**1**	0	**1**	0	0	0	0	0	0	**6**
**Other**	0	**3 (MIP), 1 (competence), 1 (Wzy_C), 1 (FTSW_RODA_SPOVE), 1 (citMHS)**	**1 (Glyco_tranf_2_3)**	0	0	0	0	0	0	0	0	**3 (MIP). 2 (7tm_1), 1 (SNARE), 1 (E1-E2_ATPase)**
**Unknown**	0	**1835**	**5**	0	**1**	0	0	0	0	0	**8**	**2220**

Table S4. The 9 Pfam families that constitute the Pfam Acyl_transf_3 Clan (CL0316). Pfam families where members are included in TCDB are mentioned with their TC code. Since the submission of our preprint to bioRxiv (11/11/24), the clan has been revised to match our definition of the TmAT superfamily which includes HGSNAT, AT3, DUF5009, DUF418, OpgC-C, DUF418, TraX, Cas1_AcylT and GWT1.

Pfam family	Name	TCDB	TmAT	Example
PF01757	Acyl_transf_3 (AT3)	9.B.97	Yes	*S.* Typhimurium OafB
PF04235	DUF418	9.B.169	Yes	*E. coli* YeiB
PF07786	HGSNAT-cat	9.B.169	Yes	Human HGSNAT structures
PF05857	TraX[1]	N/A	Yes*	*E. coli* TraX (plasmid bourne)
PF07779	Cas1_AcylT	N/A	Yes	*Cryptococcus neoformans* Cas1p
PF10129	OpgC-C	N/A	Yes	*Ricinus communis* OpgC
PF11318	DUF3120[2]	N/A	No	*Synechocystis* sp. Slr1990
PF12291	DUF3623[3]	N/A	No	*R. capsulatus* RCAP_rcc00656
PF16401	DUF5009[4]	9.B.169	Yes	*B. thetaiotaomicron* BT_0446

[1] TraX has 9 TMH in the fold, missing the usual TM10. Also, TM9 is not broken. Some of the TraX proteins included in this analysis do have 10TM helices (Uniprot ID: A0A653CKF7 and P22709) but the extra helix comes before the TmAT-defined TM1. Additionally, another couple of TraX proteins have a short TM1 (Uniprot ID: A0A497P6U4 and A0A077ZID6). There is a conserved Kx_9DH motif in what would be TM1 and other helices provide conserved R residues on the potential acyl-CoA binding face, so there are certainly features that are conserved. Given its known biological function in acetylation, we support the hypothesis that this is a divergent member of the superfamily. Its divergence is perhaps related to its acceptor substrate which is thought to be an Alanine residue in the F-pilin protein and not a sugar (26).

[2]DUF3120 are shorter proteins than typical TmAT proteins, at around 200 amino acids and contain 6 TMH. Foldseek does not find significant matches to any known TmAT proteins, nor in fact, any confident matches to any other known protein fold.

[3] DUF3623 are around 280 amino acids, so shorter than regular TmAT proteins and are predicted to have 7 TMH. As for DUF3120, Foldseek does not find significant matches to any known TmAT proteins, nor in fact any confident matches to any other known fold.

[4]DUF5009 is similar to HGSNAT, although it has two sets of two additional TM helices on either side of the protein. It is suggested in TCDB to be part of the HGSNAT/YeiB family (9.B.169).

Table S5. **The 55 proteins analysed within the TmAT superfamily, along with the corresponding residues to which each structure was truncated to make the sequence based phylogenetic tree.** Proteins were trimmed to their 10 transmembrane (TM) domain - where applicable - based on SSM alignment to HGSNAT. For protein families with fewer than 10 TMs (e.g., TraX with 9 TMs, DUF3623 and DUF3120 with 7 TMs), sequences were trimmed accordingly to 9 or 7 TMs. MBOAT proteins were not trimmed to 10 TMs due to poor alignment with HGSNAT and the absence of additional domains seen in other proteins; they were instead included as an outgroup.

Protein	Residues *
HGSNAT_Euk_H.sapiens_HGSNAT_8jkv	266-663
HGSNAT_Euk_M.musculus_HGSNAT_Q3UDW8	257-656
HGSNAT_Bac_A.baumannii_A0A009IMT5	1-381 (uncut)
HGSNAT_Bac_B.fragilis_A0A015X8J3	1-387 (uncut)
HGSNAT_Arc_M.formicicum_K2QAW2	1-382 (uncut)
DUF418(YeiB)_Bac_E.coli_YeiB_P25747	1-385 (uncut)
DUF418(YeiB)_Bac_B.subtilis_YxaH_P42107	1-402 (uncut)
DUF418(YeiB)_Bac_B.acidopullulyticus_YAMY_P32819	1-346 (uncut)
DUF418(YeiB)_Arc_H.tropicale_A0A0M9ANC9	1-400 (uncut)
DUF418(YeiB)_Arc_N.gregoryi_A0A1I3PM34	1-437 (uncut)
AT3_Euk_C.elegans_NRF6_Q09225	372-822
AT3_Bac_E.coli_WecH_P37669	1-331 (uncut)
AT3_Bac_S.epidermidis_IcaC_Q5HKP7	1-355 (uncut)
AT3_Bac_S.paratyphi_OafB_A0A0H2WM30	1-343
AT3_Arc_M.thermophila_A0A0E3H889	1-431 (uncut)
OpgC-C_Bac_Arthobacter.sp. _A0A0L1LJM8	408-795
OpgC-C_Bac_P.phenanthrenivorans _A0A0B4DB69	444-831
OpgC-C_Bac_Frigobacterium.sp. _A0A0Q5RZG6	437-824
OpgC-C_Bac_A.erythreum_A0A0U4BB22	429-811
OpgC-C_Euk_R.communis _B9T8R3	1-369 (uncut)
GWT1/PigW_Euk_M.musculus_**PigW_**Q8C398	124-503
GWT1/PigW_Euk_H.sapiens_**PigW_**Q7Z7B1	123-504
GWT1/PigW_Euk_D.discoideum_**PigW_**Q54MC0	118-492
GWT1/PigW_Euk_S.cerevisiae_**GWT1_**P47026	120-490

GWT1/PigW_Euk_C.neoformans_**GWT1**_P0CP64	219-598
Cas1AcylT_Euk_H.sapiens_**CASD1**_Q96PB1	424-766
Cas1AcylT_Euk_D.rerio_**CASD1**_Q1LW89	415-755
Cas1AcylT_Euk_M.musculus_**CASD1**_Q7TN73	424-766
Cas1AcylT_Euk_C.neoformans_**Cas1**_P0CM57	488-820
Cas1AcylT_Euk_A.thaliana_**RWA4**_Q9FXG3	169-509
TraX_Euk_C.maculatus_A0A653CKF7	24-245
TraX_Euk_O.oligospora_A0A6G1MJD2	74-309
TraX_Bac_E.coli_**TraX**_P22709	27-248
TraX_Bac_R.prowazekii_**RP591**_Q9ZCW3	1-248 (uncut)
TraX_Arc_C.Bathyarchaeota_A0A2U0RV69	1-225 (uncut)
DUF3120_Euk_P.micropora_A0A1L5YBL8	14-244
DUF3120_Bac_Fischerella.sp._A0A0S3TV71	1-215 (uncut)
DUF3120_Bac_Tolypothrix.sp._A0A0D6KS64	39-259
DUF3120_Bac_P.marinus_A0A0A2CA37	1-212 (uncut)
DUF3120_Bac_T.campylonemoides_A0A0C2Q1I8	25-222
DUF3623_Euk_S.necroappetens_A0A812YP02	379-800
DUF3623_Bac_A.spongiae_A0A3N5DJ92	1-239
DUF3623_Bac_R.mucosus_A0A0A0HGM1	1-270 (uncut)
DUF3623_Bac_R.capsulatus_P26158	1-274 (uncut)
DUF3623_Bac_G.phototrophica_A0A143BHS6	32-301
MBOAT_Euk_H.sapiens_**PORCN**_7ura	1-461 (uncut)
MBOAT_Euk_H.sapiens_**HHAT**_7mhy	1-493 (uncut)
MBOAT_Bac_S.thermophilus_**DltB**_6bug	1-415 (uncut)
MBOAT_Bac_H.pylori_**PatA**_O25526	1-527 (uncut)
MBOAT_Arc_C.woesearchaeota_A0A7J4V4K0	1-409 (uncut)

*These residues are inclusive. If a protein already contained 10TMH and was not truncated, it is marked as (uncut).

Table S6. DALI structural alignment statistics of the Pfam Acyl_transf_3 Clan (CL0316), GWT1/PIG-W family and MBOAT superfamily members when compared to HGSNAT (PDB ID: 8JKV)..

Protein	Z score	RMSD	Aligned residues	Total no. of residues	Sequence ID %
HGSNAT_Euk_H.sapiens_HGSNAT_8jkv	69.0	0.0	391	391	100
HGSNAT_Euk_M.musculus_HGSNAT_Q3UDW8	62.4	0.8	390	397	88
DUF5009_Euk_P.gosspiella_A0A1E1WPQ5	55.4	1.3	384	397	43
DUF5009_Euk_B.meristosporus_A0A1Y1X0E5	41.7	2.3	345	399	32
HGSNAT_Bac_B.fragilis_A0A015X8J3	41.0	2.1	335	387	30
HGSNAT_Arc_M. formicicum_K2QAW2	40.7	2.1	335	382	29
DUF5009_Bac_F.hydatis_A0A086A418	40.5	2.3	338	379	30
DUF5009_Bac_B.fragilis_A0A016APS9	35.7	2.7	328	375	28
DUF5009_Bac_B.thetaiotaomicron_Q8AAL8	33.3	2.8	314	374	22
GWT1/PigW_Euk_M.musculus_PigW_Q8C398	28.7	3.1	316	380	13
GWT1/PigW_Euk_S.cerevisiae_GWT1_P47026	28.5	3.3	318	371	15
GWT1/PigW_Euk_H.sapiens_PigW_Q7Z7B1	28.4	3.1	315	382	13
GWT1/PigW_Euk_D.discoideum_PigW_Q54MC0	28.1	3.1	313	375	12
GWT1/PigW_Euk_C.neoformans_GWT1_P0CP64	27.6	3.5	311	380	12
HGSNAT_Bac_A.baumannii_A0A009IMT5	26.8	3.0	297	381	16
OpgC-C_Bac_P.phenanthrenivorans _ A0A0B4DB69	21.7	3.9	293	388	13
OpgC-C_Bac_Arthobacter.sp. _A0A0L1LJM8	21.5	3.9	291	388	12
OpgC-C_Bac_A.erythreum_A0A0U4BB22	21.2	3.5	279	383	11
DUF418(YeiB)_Bac_B.subtilis_YxaH_P42107	21.0	3.9	282	402	15
OpgC-C_Bac_Frigobacterium.sp. _A0A0Q5RZG6	20.9	3.7	281	388	10
OpgC-C_Euk_R.communis _B9T8R3	20.6	4.1	282	369	15
DUF418(YeiB)_Arc_H.tropicale_A0A0M9ANC9	19.5	4.5	281	400	15
DUF418(YeiB)_Bac_E.coli_YeiB_P25747	19.3	3.9	279	385	16
DUF418(YeiB)_Bac_B.acidopullulyticus_YAMY_ P32819	18.3	3.8	263	346	16
DUF418(YeiB)_Arc_N.gregoryi_A0A1I3PM34	18.2	4.6	291	437	13
AT3_Bac_S.epidermidis_IcaC_Q5HKP7	18.1	4.7	286	355	10
AT3_Arc_M.thermophila_A0A0E3H889	15.7	4.4	273	431	14
Cas1AcylT_Euk_C.neoformans_Cas1_P0CM57	15.4	4.7	264	333	8
Cas1AcylT_Euk_H.sapiens_CASD1_Q96PB1	15.3	4.7	263	343	13
AT3_Euk_C.elegans_NRF6_Q09225	15.2	4.8	291	451	13

Cas1AcylT_Euk_M.musculus_CASD1_Q7TN73	15.1	4.9	258	343	12
Cas1AcylT_Euk_A.thaliana_RWA4_Q9FXG3	14.9	4.6	262	341	10
Cas1AcylT_Euk_D.rerio_CASD1_Q1LW89	14.9	4.7	256	341	10
AT3_Bac_E.coli_WecH_P37669	13.7	5.4	249	331	13
AT3_Bac_S.paratyphi_OafB_A0A0H2WM30	13.2	4.8	245	343	11
TraX_Bac_R.prowazekii_RP591_Q9ZCW3	10.2	4.4	186	248	13
TraX_Euk_O.oligospora_A0A6G1MJD2	9.4	4.1	175	236	16
TraX_Arc_C.Bathyarchaeota_A0A2U0RV69	8.6	3.9	160	225	10
TraX_Euk_C.maculatus_A0A653CKF7	8.2	3.9	159	222	13
TraX_Bac_E.coli_TraX_P22709	7.9	4.0	169	222	13
DUF3120_Euk_P.micropora_A0A1L5YBL8	7.3	5.5	140	231	8
DUF3120_Bac_T.campylonemoides_A0A0C2Q1I8	6.4	4.7	141	222	7
DUF3120_Bac_Fischerella.sp._A0A0S3TV71	5.9	5.1	137	215	7
DUF3623_Bac_A.spongiae_A0A3N5DJ92	5.6	4.0	127	239	6
DUF3120_Bac_Tolypothrix.sp._A0A0D6KS64	5.6	4.8	160	221	4
DUF3120_Bac_P.marinus_A0A0A2CA37	5.4	5.6	161	212	9
DUF3623_Bac_R.capsulatus_P26158	5.1	4.3	148	274	10
DUF3623_Bac_R.mucosus_A0A0A0HGM1	5.1	6.8	99	270	8
MBOAT_Bac_H.pylori_PatA_O25526	5.1	4.8	115	527	10
DUF3623_Bac_G.phototrophica_A0A143BHS6	4.9	4.4	133	270	9
DUF3623_Euk_S.necroappetens_A0A812YP02	4.7	4.5	151	263	10
MBOAT_Euk_H.sapiens_PORCN_7ura	4.5	6.2	124	432	7
MBOAT_Arc_C.woesearchaeota_A0A7J4V4K0	3.9	7.4	84	409	8
MBOAT_Euk_H.sapiens_HHAT_7mhy	3.5	5.9	105	489	10
MBOAT_Bac_S.thermophilus_DltB_6bug	3.2	4.0	74	414	4

Table S7. The 6 conserved residues of the TmAT superfamily (along with the partially conserved TMH9-Ser and TMH3-Arg) are listed with their corresponding residue numbers and respective helices in both HGSNAT and OafB.

**TMH-residue**	**HGSNAT**	**OafB**
TMH1-Asp	272	11
TMH1-Arg	275	14
TMH2-Phe	313	41
TMH2-Gly	318	46
TMH3-Arg	345	71
TMH6-Gly	514	207
TMH9-Tyr	611	292
TMH9-Ser	607	288
