# Supplementary material for: Structural unification of diverse transmembrane acyltransferases reveals a conserved fold for the transmembrane acyl transferase (TmAT) superfamily
Source: J Biol Chem. 2025 Aug 5;301(9):110546. doi: 10.1016/j.jbc.2025.110546 (PMC12423673; doi:10.1016/j.jbc.2025.110546)
Supplement: Fig S12 [file mmc2.pdf]

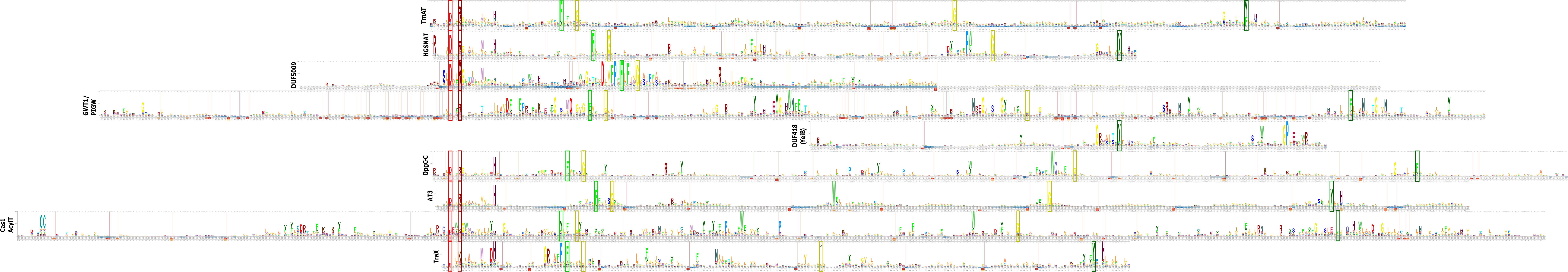
TmAT
HGSNAT
DUF5009
GWT1/PIGW
DUF418 (YeiB)
OpgC
AT3
Cas1 AcylT
TraX
