# Supplementary material for: Structural unification of diverse transmembrane acyltransferases reveals a conserved fold for the transmembrane acyl transferase (TmAT) superfamily
Source: J Biol Chem. 2025 Aug 5;301(9):110546. doi: 10.1016/j.jbc.2025.110546 (PMC12423673; doi:10.1016/j.jbc.2025.110546)
Supplement: Fig S13 [file mmc3.pdf]

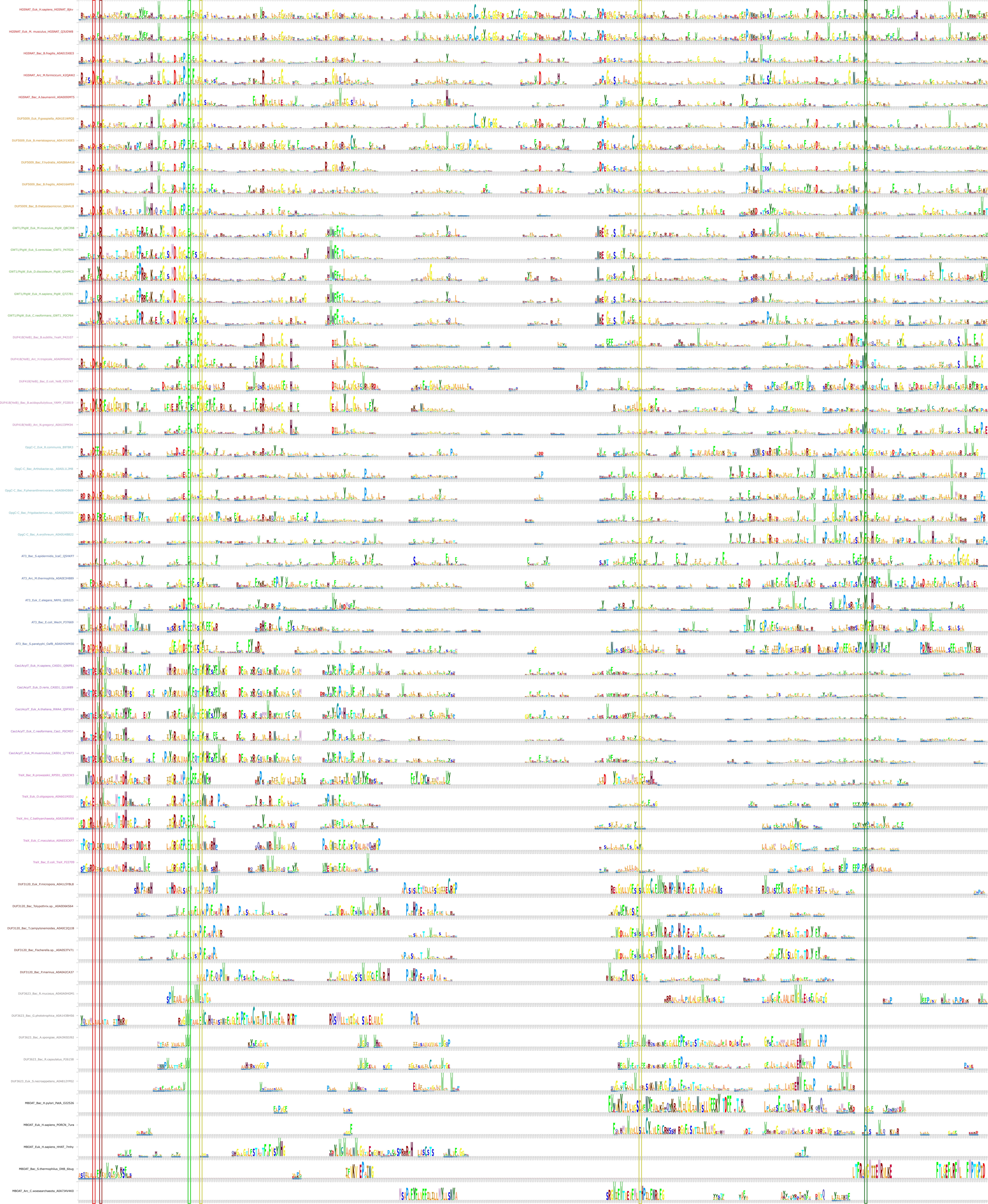

HGSNAT_Euk_H.sapiens_HGSNAT_8jkv
HGSNAT_Euk_M. musculus_HGSNAT_Q3UDW8
HGSNAT_Bac_B.fragilis_A0A015X8J3
HGSNAT_Arc_M.formicicum_K2QAW2
HGSNAT_Bac_A.baumannii_A0A009IMT5
DUF5009_Euk_P.gosspiella_A0A1E1WPQ5
DUF5009_Euk_B.meristosporus_A0A1Y1X0E5
DUF5009_Bac_F.hydratis_A0A086A418
DUF5009_Bac_B.fragilis_A0A016APS9
DUF5009_Bac_B.thetaiotaomicron_Q8AAL8
GWT1/PigW_Euk_M.musculus_PigW_Q8C398
GWT1/PigW_Euk_S.cerevisiae_GWT1_P47026
GWT1/PigW_Euk_D.discoideum_PigW_Q54MC0
GWT1/PigW_Euk_H.sapiens_PigW_Q7Z7B1
GWT1/PigW_Euk_C.neoformans_GWT1_P0CP64
DUF418(YeiB)_Bac_B.subtilis_YxaH_P42107
DUF418(YeiB)_Arc_H.tropicale_A0A0M9ANC9
DUF418(YeiB)_Bac_E.coli_YeiB_P25747
DUF418(YeiB)_Bac_B.acidopullulyticus_YAMY_P32819
DUF418(YeiB)_Arc_N.gregoryi_A0A1I3PM34
OpgC-C_Euk_R.communis_B9T8R3
OpgC-C_Bac_Arthobacter.sp._A0A0L1LJM8
OpgC-C_Bac_P.phenanthrenivorans_A0A0B4DB69
OpgC-C_Bac_Frigobacterium.sp._A0A0Q5RZG6
OpgC-C_Bac_A.erythreum_A0A0U4BB22
AT3_Bac_S.epidermidis_IcaC_Q5HKP7
AT3_Arc_M.thermophila_A0A0E3H889
AT3_Euk_C.elegans_NRF6_Q09225
AT3_Bac_E.coli_WecH_P37669
AT3_Bac_S.paratyphi_OafB_A0A0H2WM30
Cas1AcylT_Euk_H.sapiens_CASD1_Q96PB1
Cas1AcylT_Euk_D.rerio_CASD1_Q1LW89
Cas1AcylT_Euk_A.thaliana_RWA4_Q9FXG3
Cas1AcylT_Euk_C.neoformans_Cas1_P0CM57
Cas1AcylT_Euk_M.musmculus_CASD1_Q7TN73
TraX_Bac_R.prowazekii_RP591_Q9ZCW3
TraX_Euk_O.oligospora_A0A6G1MJD2
TraX_Arc_C.bathyarchaeota_A0A2U0RV69
TraX_Euk_C.maculatus_A0A653CKF7
TraX_Bac_E.coli_TraX_P22709
DUF3120_Euk_P.micropora_A0A1L5YBL8
DUF3120_Bac_Tolypothrix.sp._A0A0D6KS64
DUF3120_Bac_T.campylonemoides_A0A0C2Q1I8
DUF3120_Bac_Fischerella.sp._A0A0S3TV71
DUF3120_Bac_P.marinus_A0A0A2CA37
DUF3623_Bac_R.mucosus_A0A0A0HGM1
DUF3623_Bac_G.phototrophica_A0A143BHS6
DUF3623_Bac_A.spongiae_A0A3N5DJ92
DUF3623_Bac_R.capsulatus_P26158
DUF3623_Euk_S.necroappetens_A0A812YP02
MBOAT_Bac_H.pylori_PatA_O22526
MBOAT_Euk_H.sapiens_PORCN_7ura
MBOAT_Euk_H.sapiens_HHAT_7mhy
MBOAT_Bac_S.thermophilus_DltB_6bug
MBOAT_Arc_C.woesearchaeota_A0A7J4V4K0
